# Migration and biotransformation mechanisms of risk-priority antibiotics in wastewater biotreatment: An integrated multi-omics and molecular dynamics perspective

**DOI:** 10.1016/j.eehl.2026.100260

**Published:** 2026-06-25

**Authors:** Bingqing Wang, Zuxin Xu, Bin Dong

**Affiliations:** a State Key Laboratory of Pollution Control and Resource Reuse, College of Environmental Science and Engineering, Tongji University, Shanghai, 200092, China; b Ministry of Education Key Laboratory of Yangtze River Water Environment, Tongji University, Shanghai, 200092, China

**Keywords:** Antibiotic biotransformation, Mixed antibiotic exposure, Ciprofloxacin (CIP), Sulfamethoxazole (SMX), Roxithromycin (ROX), Metagenomics, Metaproteomics, Activated sludge

## Abstract

Understanding the fate and transformation of antibiotics is essential for controlling antibiotic pollution in wastewater treatment plants (WWTPs). This study integrated metagenomics, metaproteomics, molecular dynamics (MD) simulations, and pathway analysis to elucidate the behavior of ciprofloxacin (CIP), sulfamethoxazole (SMX), and roxithromycin (ROX) under single- and mixed-antibiotic exposures in an activated sludge system. Fate analysis revealed divergent pathways: SMX was predominantly biodegraded (>70%), whereas CIP and ROX were mainly adsorbed onto sludge, showing poor removal and high effluent residuals (CIP > 50%, ROX > 60%). Under mixed-antibiotic stress, microorganisms favored lower-energy degradation pathways, leading to simplified (skip-step) transformations. MD simulations unveiled that within the extracellular polymeric substances (EPS) matrix, the protein fraction exhibited the strongest binding. Docking and MD simulations on a proteomics-derived interface-associated protein (OmpA) revealed a co-adsorption behavior under mixed-antibiotic exposure, where CIP strongly anchored through multipoint hydrogen bonding/electrostatic interactions and facilitated SMX stabilization in the same pocket via aromatic stacking. Multi-omics analyses revealed a microbial “survival-first” strategy dominated by resistance and repair. Notably, transporter-related stress responses were prominent under mixed stress, and several ABC transporter-associated components (e.g., K02003/K02004 and K02033) were negatively correlated with removal efficiency, coinciding with reduced degradation by key genera such as *Micropruina* and *Ottowia*. Under mixed-antibiotic stress, a confluence of reinforced resistance (e.g., Type IV secretion system K03205), altered EPS binding, and skewed energy allocation (e.g., downregulation of cofactor synthesis ko01240) led to incomplete degradation and widespread persistence. This study provides a multiscale theoretical framework for optimizing WWTPs to control antibiotic pollution.

## Highlights

• Elucidated phase partitioning and long-term fate of eco-risky antibiotics.
• Mixed exposure simplified degradation with skip-step routes of lower energy barriers.
• MD confirmed EPS proteins dominate adsorption and enabled synergistic co-adsorption.
• Identified survival-first response driving higher residues under mixed antibiotics.
• Multi-omics analysis linked microbial adaptation to antibiotic persistence.

## Graphical abstract

**Figure:**
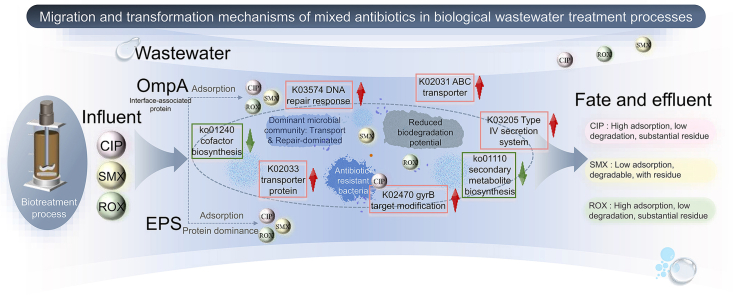

## 1 Introduction

Antibiotics, indispensable in modern medicine and animal husbandry, have emerged as ubiquitous contaminants frequently detected in aquatic environments due to their extensive use, incomplete metabolism, and inherent recalcitrance [1]. Antibiotic pollution, coupled with the potential risk of antimicrobial resistance (AMR), poses a significant threat to ecosystem stability and human health [2]. Wastewater treatment plants are recognized as hotspots for the accumulation of antibiotics [3]. Their biological treatment units, while designed for conventional pollutant removal, also serve as critical junctures controlling the environmental fate of antibiotics. Numerous studies have demonstrated that antibiotics can persist long-term in these systems, exerting profound impacts on effluent water quality and the dissemination of antibiotic resistance genes (ARGs) [4].

Continuous exposure of activated sludge systems to antibiotics often leads to the remodeling of microbial community structures, shifts in the abundance of functional genes, and compromised removal efficiency of nutrients such as nitrogen and phosphorus [5]. The inadequate removal efficacy of existing WWTP processes against antibiotics results in the continuous discharge of both parent compounds and their transformation products into aquatic ecosystems [6]. During biological wastewater treatment, antibiotics not only persist as parent compounds but also undergo complex processes including adsorption, biodegradation, and conversion into intermediate products. These dynamic behaviors directly determine their environmental fate and potential risks [7]. Therefore, investigating the migration and transformation patterns of antibiotics in WWTPs is crucial not only for assessing their persistence and ecological effects but also for providing a scientific basis for process optimization.

Previous studies have explored the adsorption and degradation pathways of antibiotics in activated sludge systems and revealed their impacts on microbial communities and ARG propagation. For instance, Peng et al. [8] reported the adsorption and transformation behaviors of various pharmaceuticals in WWTPs, finding that for six biodegradable drugs, heterotrophic biodegradation and adsorption were the primary removal routes. Li et al. [9] reported that norfloxacin could interfere with the activity of nitrifying bacteria, leading to a decline in nitrogen transformation efficiency, while research by Li et al. [10] indicated that sulfonamides [e.g., sulfamethoxazole (SMX)] could influence ARG dissemination by altering key metabolic pathways. However, the co-existence of multiple antibiotics, a common scenario in real-world environments, can trigger complex synergistic or antagonistic effects, and the underlying mechanisms governing these interactions and their impacts on transformation processes remain insufficiently understood. Yet, most research has been limited to single-antibiotic systems or short-term exposure [11,12], and therefore has not sufficiently resolved the mechanistic basis of antibiotic behavior under realistic, mixed-contaminant conditions. Furthermore, current research predominantly focuses on macroscale removal efficiency and risk assessment [13], while mechanistic understanding linking molecular interactions, transformation processes, and microbial responses remains limited. A systematic investigation from a molecular perspective, particularly one that elucidates the interactions between antibiotics and extracellular polymeric substances (EPS) and their regulatory effects on transformation pathways, remains largely lacking. This knowledge gap constrains a deeper understanding of the environmental fate and ecological risks of antibiotics, highlighting the urgent need for a comprehensive elucidation to support advanced wastewater treatment and resistance control.

To address these gaps, this study employed long-term reactor operation combined with multi-omics and molecular simulation approaches to investigate antibiotic migration and transformation from a mechanistic perspective, linking molecular interactions, transformation pathways, and microbial community responses under both single and mixed-antibiotic stress. This study aims to provide mechanistic insights to support the optimization of wastewater treatment processes and the control of antibiotic pollution. Specifically, we selected three antibiotics with moderate to high ecological risks in both effluent and sludge of WWTPs [14], that is, ciprofloxacin, sulfamethoxazole, and roxithromycin. Over a 200-day operation of sequencing batch reactors, we systematically analyzed their long-term fate, phase partitioning, and biotransformation mechanisms under single or mixed-antibiotic stress. To achieve this, a multiscale approach was adopted: metagenomic pathway enrichment analysis was used to identify key pathways under single or mixed exposure to ciprofloxacin (CIP), SMX, and roxithromycin (ROX); differential expression analysis and species contribution based on the Kyoto Encyclopedia of Genes and Genomes (KEGG) Orthology (KO) system were used to identify functional genera and core resistance/degradation genes; and molecular dynamics (MD) simulations combined with density functional theory (DFT) were applied to elucidate the molecular mechanisms and thermodynamic preferences of antibiotic binding to EPS proteins. The results provide a new molecular perspective on the mechanisms behind the partial degradation and widespread persistence of antibiotics, offering a theoretical basis for optimizing wastewater treatment processes and mitigating environmental resistance risks.

## 2 Materials and methods

### 2.1 The bioreactors and operation

To simulate the biological wastewater treatment process, five sequencing batch reactors (SBRs), each with a working volume of 4 L, were established. These reactors were designated as the control group, the CIP group, the SMX group, the ROX group, and a mixed-antibiotic group. The initial activated sludge was collected from the secondary clarifier of a municipal wastewater treatment plant in Shanghai, China. The seed sludge exhibited the following characteristics: mixed liquor suspended solids (MLSS) ranging from 2920 to 3250 mg/L, a mixed liquor volatile suspended solids to MLSS ratio (MLVSS/MLSS) of 79% to 82%, and a pH of 6.85 to 7.10.

All five reactors were configured to achieve simultaneous carbon removal and biological nitrogen and phosphorus removal. They were operated as SBRs under identical conditions [two 12-h cycles per day; hydraulic retention time (HRT) ≈ 1 d; solids retention time (SRT) = 20 d]. Reactors were run at room temperature without external temperature or pH control; dissolved oxygen (DO) was maintained at 0.16–0.24 mg/L during mixing, and aeration was supplied (about 1.0 L/min) and regulated using a gas flow meter. Full operational details are provided in Table S1 and Text S1.

After start-up, the reactors were fed with synthetic wastewater designed to simulate typical municipal wastewater composition. This approach was adopted to minimize the variability associated with real wastewater and to ensure comparability among different treatment conditions. The synthetic influent contained sodium acetate, glucose, and sucrose as carbon sources, ammonium chloride as the nitrogen source, and potassium dihydrogen phosphate as the phosphorus source. Detailed composition is provided in Table S2.

After a 20-day acclimation period without antibiotics, CIP, SMX, and ROX were introduced into the reactors. In the single-antibiotic reactors, each antibiotic was added at an influent concentration of 1.0 mg/L. In the mixed reactor, the three antibiotics were applied simultaneously at a ratio of 1:1:1, with 1.0 mg/L for each compound (total 3.0 mg/L), ensuring consistent individual concentrations across treatments. At the beginning of each cycle, influent was fed for 30 min with a volume of 2 L per cycle. Antibiotics were dissolved together with nutrients (C, N, and P) in the influent and introduced during the feeding phase. Therefore, antibiotic dosing was performed as cycle-based influent dosing, rather than pulse shock or continuous drip addition. The selected concentration (1.0 mg/L) represents a mechanism-oriented stress level for investigating transformation pathways and interaction effects under controlled conditions.

### 2.2 Physicochemical analysis

Influent, effluent, and sludge samples were regularly collected from the reactors to determine conventional water quality parameters and antibiotic concentrations. Prior to analysis, all aqueous samples were filtered through 0.22 μm membrane filters. The concentrations of chemical oxygen demand (COD), total nitrogen (TN), total phosphorus (TP), ammonia-nitrogen (NH_4_^+^-N), nitrate-nitrogen (NO_3_^−^-N), and nitrite-nitrogen (NO_2_^−^-N) were measured according to the standard methods [15].

Extracellular polymeric substances were extracted following the method described in Ref. [16]. Subsequently, the contents of polysaccharides (PS) and proteins (PN) in the EPS were determined using the phenol-sulfuric acid method and the Lowry method, respectively [17]. FTIR spectra were collected using a Nicolet iS20 FTIR spectrometer (Thermo Fisher Scientific) over 400–4000 cm^−1^ with 32 scans at 4 cm^−1^ resolution. The Excitation-Emission Matrix (EEMs) were measured using a fluorescence spectrometer (Hitachi FL-7100, Japan) and the obtained EEM data were preprocessed as follows: (1) subtracting the background fluorescence of ultrapure water; (2) clipping Raman and Rayleigh scattering and smoothing with interpolation using the Dreem toolbox in MATLAB [18]. The concentrations of antibiotics in the influent, effluent, and EPS were quantified using a high-performance liquid chromatography-mass spectrometry (HPLC-MS) system (Shimadzu LC-MS 2050). The intermediate products of antibiotic biodegradation were detected by TOF-MS (Agilent 6550 Q-TOF coupled with Agilent 1290 UPLC). Based on these measurements, the fates of the three antibiotics during the stable operational phase (day 110 of operation) were calculated via a mass balance approach. Details regarding physicochemical analysis are provided in Text S1.

### 2.3 Molecular docking and molecular dynamics simulation

#### 2.3.1 Computational methods for degradation pathway analysis

Details are provided in Text S2.

#### 2.3.2 Computational methods for protein docking

Small molecules (antibiotics and the TNB humic-acid model) were optimized in ORCA [B3LYP-D3(BJ)/6-311G(d,p)]; RESP charges were obtained with Multiwfn. GAFF (Sobtop) and Amber14SB (GROMACS) were used for topology generation. EPS simulations were built with Packmol in a 12 nm cubic box, while OmpA–antibiotic complexes were solvated in TIP3P water (1.5 nm padding) with NaCl neutralization. The OmpA structure used in this study was obtained from the AlphaFold Protein Structure Database (AF-Q05146-F1-v6). Only the soluble domain (residues 77−193) was retained for simulation, while the transmembrane region was excluded to focus on the accessible domain involved in interfacial interactions. No manually selected template was used in this workflow. The composite EPS matrix was used as a representative minimal model rather than a full structural reconstruction of natural sludge EPS. The three components were spatially assembled in the simulation box and subsequently relaxed by energy minimization and equilibration, rather than being treated as a pre-formed chemically bonded complex. All MD simulations employed steepest-descent energy minimization, 0.9 nm real-space cutoffs, Particle Mesh Ewald (PME) electrostatics, and production runs in the constant number of particles, pressure, and temperature (NPT) ensemble at 298.15 K/1 bar for 100 ns (EPS) and 200 ns (OmpA). Detailed MD settings are provided in Text S3.

### 2.4 Bioinformatic and statistical analysis

Metagenomic reads were quality-filtered with fastp and assembled using MEGAHIT. Genes were predicted with Prodigal (≥100 bp) and clustered with CD-HIT to construct a non-redundant catalog. Reads were mapped using SOAPaligner and gene abundance normalized by RPKM. Functional annotation was performed against the KEGG and NR databases. Metagenomic libraries were sequenced on an Illumina NovaSeqTM X Plus platform using paired-end 150 bp reads, generating approximately 6–10 Gb of clean data per sample. Five independent SBRs were operated, corresponding to the control, CIP, SMX, ROX, and mixed-antibiotic conditions, with one reactor per condition. Samples collected at different time points from the same reactor were treated as longitudinal/time-series samples rather than independent biological replicates. Raw metagenomic reads were processed using the Majorbio standardized pipeline. Group-wise comparisons were performed using platform-implemented statistical modules, with non-parametric tests applied where appropriate and false discovery rate (FDR) correction used for multiple testing. Statistical significance was evaluated based on FDR-adjusted *p* values, as specified for each analysis.

Protein identification was accepted at Q-value <0.01, quantification was based on high-confidence peptides, and differential proteins were defined as |log_2_fold change| > 1, FDR-adjusted *p* < 0.05. Detailed information is provided in Text S4. For metaproteomic analysis, total proteins were extracted from sludge samples using a phenol-based protocol, followed by tryptic digestion. LC-MS/MS data were acquired in data-independent acquisition (DIA) mode on an Orbitrap Astral mass spectrometer. Protein identification was performed against a non-redundant protein/gene catalog constructed from the metagenomic dataset generated in this study, thereby ensuring consistency between metagenomic and metaproteomic analyses. DIA raw data were processed using Spectronaut (version 19), with trypsin/P specificity, up to 2 missed cleavages, carbamidomethylation of cysteines as a fixed modification, oxidation of methionines and protein N-terminal acetylation as variable modifications, and FDR controlled at ≤1% at both protein and peptide levels.

## 3 Results and discussion

### 3.1 Treatment performance and operational stability under antibiotic exposure

**Fig. 1:**
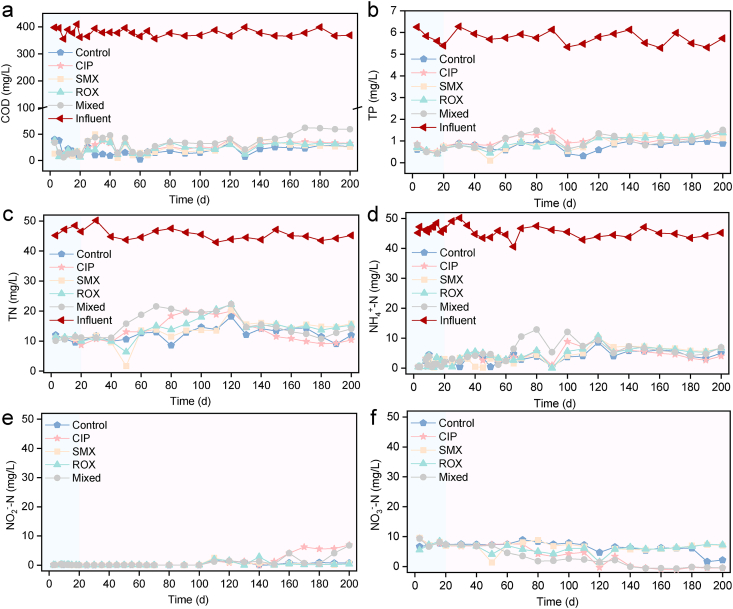
Long-term monitoring of effluent concentrations in the reactors: (a) COD, (b) TP, (c) TN, (d) NH_4_^+^-N, (e) NO_2_^−^-N, and (f) NO_3_^−^-N. CIP, ciprofloxacin; SMX, sulfamethoxazole; ROX, roxithromycin; Mixed, mixed-antibiotic treatment containing CIP, SMX and ROX.

Understanding the microbial functional responses to antibiotic stress is fundamental to assessing the stability of treatment performance and the risks associated with antibiotic migration in biological wastewater treatment systems. By monitoring the temporal changes of six water quality indicators (Fig. 1a–f), we observed that during the initial antibiotic-free period (0–20 d), all reactors rapidly achieved stable and efficient removal of conventional pollutants after a brief acclimation phase. However, following the introduction of antibiotic stress (from day 20 onward), the reactors exhibited slight variations in treatment performance. Overall, the control reactor maintained stable performance, providing a benchmark for the treatment groups. While the single-antibiotic groups showed a slight decline in performance, the mixed-antibiotic group exhibited the most noticeable, yet still moderate, changes among all treatments. Throughout the entire operational period, the effluent concentrations of all reactors consistently met the relevant national discharge standards for municipal wastewater treatment (Class 1B), confirming the successful establishment and stable operation of the biological treatment systems.

In the CIP group, TN removal decreased slightly between days 40 and 140, and NO_2_^−^-N removal decreased between days 140 and 180. Descriptive patterns of functional genes related to nitrogen and phosphorus metabolism under different antibiotic treatments are provided in Fig. S1 for qualitative reference only, without mechanistic inference. Long-term exposure to SMX resulted in a 2.0% decrease in TN removal efficiency, indicating a moderate but persistent impact on nitrogen removal performance [19]. In the ROX group, the TN and TP removal efficiencies decreased by 3.9% and 2.8%, respectively. In contrast, the mixed-antibiotic group exhibited the most pronounced changes in treatment performance among all reactors. The average removal efficiency for COD, TP, TN, and NH_4_^+^-N declined by 3.6%, 3.6%, 6.3%, and 3.1%, respectively. These results indicate that combined antibiotic exposure led to cumulative effects on overall treatment performance, while the system remained operationally stable and compliant with discharge standards.

### 3.2 Fate of antibiotics

**Fig. 2:**
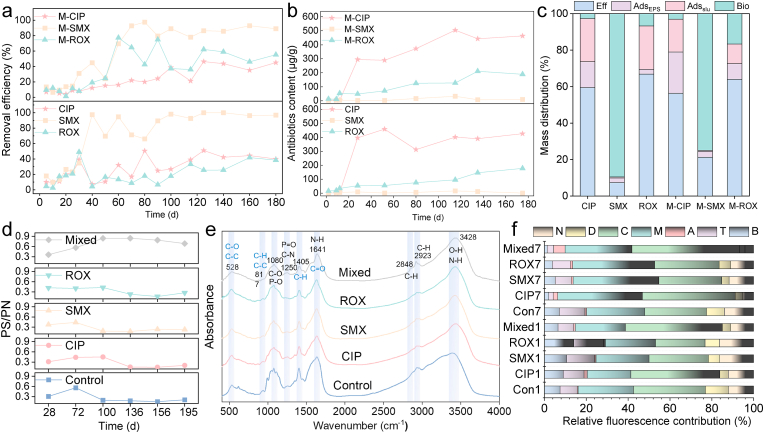
Long-term monitoring of (a) aqueous phase removal efficiency and (b) sludge accumulation of antibiotics in the single- and mixed-antibiotic groups. (c) Fate of antibiotics in the single- and mixed-antibiotic groups. (d) Long-term monitoring of the polysaccharide-to-protein ratio (PS/PN). (e) FTIR spectra of EPS from each group. Key FTIR bands were assigned as follows: 1641 cm^−1^ (amide I, protein), 3428 cm^−1^ (O–H stretching, hydroxyl-related adsorption sites), 1080 cm^−1^ (C–O–C stretching, polysaccharides), and 1405 cm^−1^ (COO^−^ stretching, uronic-acid-related groups). (f) Peak-picking results from 3D-EEM for each group at the initial (1) and late (7) stages of antibiotic exposure. For 3D-EEM, peak B and peak T represent tyrosine-like and tryptophan-like proteinaceous substances, respectively; peak N represents protein-like substances; and peaks D, C, M, and A represent humic-like substances. 3D-EEM, three-dimensional excitation–emission matrix fluorescence spectroscopy; Ads_EPS_, adsorption to EPS; Ads_slu,_ adsorption to sludge; Bio, biodegradation; Con, control; Eff, effluent; EPS, extracellular polymeric substances; FTIR, Fourier transform infrared spectroscopy.

The long-term fate of antibiotics was monitored in both single- and mixed-antibiotic groups, revealing distinct removal patterns (Fig. 2a–c). In the aqueous phase, the antibiotic removal efficiency in the single-antibiotic groups gradually increased before reaching stable levels after the first 40 days, whereas the stabilization period for the mixed-antibiotic group was delayed until day 60. After day 120, the removal efficiency in all groups became largely stable, with average efficiencies following the order of SMX > ROX ≈ CIP (Fig. 2a). A similar pattern was observed in the sludge phase, where antibiotic accumulation increased steadily within the first 28 days and reached a plateau after day 115. The adsorption affinity followed the order: CIP > ROX > SMX (Figs. 2b and S2).

Integration of temporal dynamics with fate distribution analysis (Fig. 2c) revealed distinct partitioning and transformation behaviors for the three antibiotics. Based on mass balance analysis, SMX exhibited the highest removal efficiency and the lowest accumulation in sludge, indicating that its removal in the acclimated activated sludge system was predominantly driven by microbial biodegradation (>70%) [20]. Based on the operationally defined mass balance analysis separating aqueous, EPS-associated, and residual sludge-associated fractions, EPS-associated adsorption was identified as the secondary removal pathway for SMX, whereas adsorption to the residual sludge matrix after EPS extraction contributed only a minor fraction. Detailed calculations are provided in the Text S1. This behavior is attributed to the high polarity and hydrophilicity of SMX, which reduce its hydrophobic adsorption onto the sludge surface and enhance its mobility through strong interactions with water molecules [21]. Furthermore, due to competitive effects among coexisting antibiotics, the microbial degradation of SMX was compromised in the mixed group, as evidenced by its significantly higher residual concentration in the effluent (21.3%) compared to the single-antibiotic group (7.4%).

In contrast, CIP and ROX exhibited similar fates, characterized by weak biodegradation. After treatment, over 50% of CIP and over 60% of ROX remained in the effluent. The removal of both CIP and ROX was governed primarily by adsorption to EPS and sludge (Ads_eps_, Ads_slu_). Notably, EPS exhibited a higher adsorption affinity for CIP than the bulk sludge matrix did [22], whereas for ROX, the role of sludge adsorption was more pronounced than that of EPS adsorption. This is because CIP is adsorbed via combined electrostatic and hydrophobic interactions mediated by its aromatic ring, fluorine atom, and protonated piperazine ring, while ROX binds to sludge through electrostatic interactions of its protonated dimethylamino group and hydrophobic effects from its macrocyclic lactone structure.

Adsorption in sludge systems is a complex, heterogeneous process [23]. The relative contribution of proteins and polysaccharides in sludge EPS is commonly assessed using a compositional ratio. In this study, we use the polysaccharide-to-protein ratio (PS/PN). As shown in Fig. 2d, the mixed group exhibited the highest PS/PN ratio, indicating an increased relative contribution of polysaccharides in EPS under the combined antibiotic exposure. As shown in Fig. 2e, the peak at 1641 cm^−1^ is corresponding to amide groups in proteins [24], contributed most strongly to adsorption in the mixed and CIP groups. The band at 3428 cm^−1^ represents active adsorption sites provided by hydroxyl groups [25]. The peak at 1080 cm^−1^ is attributed to the C−O−C stretching vibration in polysaccharides or polysaccharide-like substances, and the band at 1405 cm^−1^ corresponds to COO^−^ stretching, which may be associated with uronic acids [26]. Additionally, three-dimensional fluorescence spectroscopy (3D-EEM) results (Fig. 2f) corroborated the involvement of protein-like substances, specifically tyrosine (peak B) and tryptophan (peak T), in the adsorption of the three antibiotics [27]. These results suggest that protein-associated functional groups within EPS play an important role in antibiotic–EPS interactions, which motivates subsequent analysis at the molecular level.

### 3.3 Mechanisms of antibiotic adsorption

#### 3.3.1 Molecular interpretation of EPS-driven antibiotic adsorption by MD

**Fig. 3:**
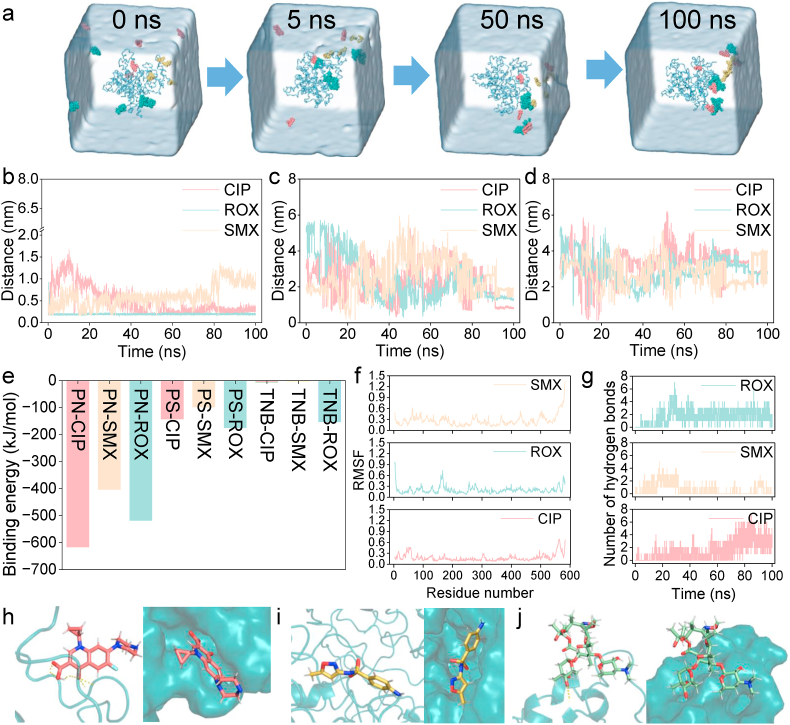
(a) Representative MD snapshots of antibiotic adsorption in the composite EPS system; minimum distances between antibiotics and EPS (b) protein, (c) polysaccharide, and (d) humic acid. (e) Binding energies between antibiotics and EPS components. (f) RMSF of the protein backbone. (g) Time evolution of hydrogen bonds between protein and antibiotics; representative binding conformations of the protein with (h) CIP, (i) SMX, and (j) ROX. MD, molecular dynamics; PN, proteins; PS, polysaccharides; TNB, humic-acid model compound; RMSF, root mean square fluctuation.

Fig. 3a presents representative MD snapshots of three antibiotics (CIP, ROX and SMX) interacting with a composite EPS matrix (protein-polysaccharide-humic acid), where only the antibiotics and the protein are shown for clarity. CIP, ROX and SMX are colored in pink, green and yellow, respectively. At 0 ns, the antibiotic molecules were randomly dispersed in the simulation box and were not yet associated with EPS components, resulting in a highly disordered configuration. By 5 ns, incipient adsorption became evident as the antibiotics approached the EPS surface, likely driven by van der Waals forces and electrostatic attraction, forming weak and transient interactions that mark the early diffusion and initial association stage. At 50 ns, adsorption was enhanced, and the antibiotics began to accumulate at specific EPS sites (e.g., hydrophobic pockets, charged residues and surface grooves on the protein), yielding more stable intermediate complexes, as reflected by shortened intermolecular distances and the emerging hydrogen-bond network. By 100 ns, pronounced antibiotic clustering around EPS was observed, driven collectively by electrostatic interactions, hydrogen bonding, π–π stacking and hydrophobic effects.

Binding free energy calculations combined with distance analyses further revealed the binding characteristics between the antibiotics and individual EPS components. As shown in Fig. 3b–d and Text S12, among the three EPS constituents, the protein exhibited the strongest interaction with all antibiotics, as indicated by the minimum protein-antibiotic distances (Fig. 3b). Consistently, the binding energies between the protein and antibiotics were markedly stronger (more favorable) than those associated with the polysaccharide or humic acid (Fig. 3e). This preferential binding is likely attributable to the presence of defined hydrophobic pockets, charged residues (e.g., arginine and lysine) and flexible loop regions on the protein surface [28], which can engage the aromatic rings, carboxyl groups or amine groups of antibiotics through hydrogen bonding, electrostatic attraction and van der Waals interactions, resulting in higher energetic contributions and lower binding free energies along the MD trajectories [29]. In contrast, antibiotic binding to the polysaccharide and humic acid was weaker (Fig. 3e), characterized by longer and more fluctuating distances (Fig. 3c–d). This can be explained by the largely linear/branched polysaccharide architecture that lacks specific binding sites and is readily perturbed by solvent competition. Humic acid, as an amorphous organic polymer, possesses heterogeneous and relatively hydrophilic surfaces, rendering antibiotic contacts more random and non-specific; consequently, large fluctuations were observed and persistent strong binding was rarely established, leading to the weakest overall interactions.

Fig. 3f–j further elucidates the protein–antibiotic interaction mechanisms. The RMSF analysis (Fig. 3f) shows that CIP binding reduced protein flexibility, ROX showed a similar trend, whereas SMX binding led to increased RMSF. These results indicate that CIP and ROX tend to form more stable complexes with the protein, thereby restricting the dynamics of specific residues/segments and increasing local rigidity and structural stability. As shown in Fig. 3g, the protein-CIP and protein-ROX systems maintained relatively high numbers of hydrogen bonds, which gradually increased or remained stable over time. Visualization of representative conformations (Fig. 3h) further suggests that CIP exhibits superior shape complementarity with surface pockets of BSA and can form multiple stable hydrogen bonds. By comparison, ROX, with its larger molecular size and flexible glycosyl side chains, suffers from pronounced steric hindrance and is less able to penetrate deep into the protein pocket (Fig. 3j), leading to weaker binding than CIP. In contrast, the SMX-protein system displayed fewer hydrogen bonds with strong fluctuations and longer binding distances, indicating loose and unstable association, likely dominated by transient interactions.

Overall, these results suggest that in wastewater treatment systems, EPS proteins are the primary components responsible for antibiotic adsorption and retention, whereas the contributions of polysaccharides and humic-like substances are comparatively minor (and may be negligible for certain antibiotics).

#### 3.3.2 OmpA-mediated adsorption and co-retention under mixed-antibiotics

To investigate protein-antibiotic interactions at the molecular level in realistic biological wastewater treatment systems, we selected a representative interface-associated protein from the proteomics dataset for molecular docking and molecular dynamics simulations. The protein candidate was prioritized based on statistical significance, biological relevance, and structural feasibility; detailed selection criteria are provided in the Text S5.

The OmpA-antibiotic complexes stabilized after approximately 100 ns. The protein backbone RMSD converged to approximately 0.30−0.35 nm (Fig. S3b), and both the ligand RMSD (relative to the protein) and the minimum protein-ligand distance became stable for all three antibiotics (Fig. S3c–d). These trends indicate that the ligands underwent a process of surface migration, site searching, and stable occupancy before adopting stable binding conformations, justifying the use of the 100−200 ns trajectories for mechanistic statistics and conformational analyses.

**Fig. 4:**
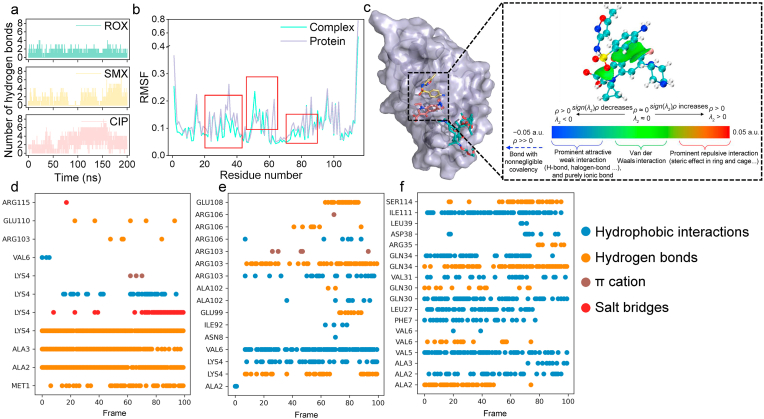
(a) The number of hydrogen bonds between antibiotics and OmpA during the molecular dynamics simulation process. (b) The RMSF changes of the protein in the OmpA-antibiotic complex and the standalone OmpA. (c) Visualization of the binding mode of antibiotics to OmpA. (d) CIP, (e) SMX, and (f) ROX interaction statistics with OmpA.

Distinct binding interactions were observed for each of the three antibiotics. CIP exhibited a shorter and less fluctuating minimum distance to OmpA and formed a richer hydrogen-bond network during the stable stage (up to approximately 6−8 H-bonds, with a pronounced increase in the mid-to-late simulation; Fig. 4a), suggesting that CIP binding is dominated by multipoint hydrogen bonding and electrostatic interactions, leading to strong adsorption and stable retention. In contrast, ROX maintained fewer hydrogen bonds over time (approximately 1–3), yet showed relatively small RMSD fluctuations, implying a binding mode more reliant on hydrophobic contacts, water-mediated bridges, and shape complementarity at a distinct site. SMX displayed a late-stage strengthening phenomenon: fewer hydrogen bonds and more pronounced conformational adjustment initially, followed by a gradual increase and stabilization of hydrogen bonds after approximately 120 ns, suggesting that its adsorption is governed more by kinetic rearrangement.

RMSF analysis (Fig. 4b) indicates that antibiotic binding did not induce large-scale conformational transitions of OmpA; however, it markedly reduced the local flexibility of specific segments (approximately residues 20−40, 45−65, and 75−90). This implies that these regions may constitute key binding/constraint elements, enhancing complex stability by reducing local conformational degrees of freedom.

Importantly, under mixed-antibiotic conditions, we observed a potentially relevant cooperative co-adsorption mechanism. Structural visualization (Fig. 4c) shows that ROX preferentially occupies a relatively independent binding site, whereas CIP and SMX can co-occupy the same pocket. Direct π–π stacking and van der Waals cooperativity between their aromatic moieties were observed, which can reduce local free energy and enhance occupancy stability. Interaction statistics further indicate that CIP interactions are more focused, forming persistent hydrogen bonds with residues such as ALA2, ALA3 and LYS4 and also exhibiting salt bridges or cation−π interactions (Fig. 4d–f). ROX interacts through a more dispersed network involving residues such as LEU39, GLN30/34, ASP38 and VAL31, consistent with a combination of hydrogen bonding/water bridges and hydrophobic contacts. SMX exhibits intermediate interaction pattern, interacting mainly with ARG103, ARG106, VAL6 and LYS4 through hydrogen bonding and hydrophobic interactions.

These findings provide a molecular explanation for differential migration and retention of structurally distinct antibiotics in sludge systems. CIP, characterized by relatively high polarity and a greater capacity for multipoint hydrogen bonding, is more readily strongly adsorbed at interface protein sites, enabling persistent retention. In contrast, SMX may exhibit weaker binding and undergo rearrangement in isolation; however, in the presence of CIP it can be co-stabilized within the same pocket through π–π cooperativity, thereby increasing the probability of co-adsorption and co-retention. This mechanism may partly explain why the average SMX adsorption in the mixed group exceeded that in the single-antibiotic group (Fig. S2). ROX, by contrast, favors an independent binding strategy primarily driven by hydrophobic and water-bridge interactions, distinct from that of CIP/SMX.

### 3.4 Proposed biodegradation pathways

To establish a molecular-scale reactivity rationale, we calculated a set of electronic-structure descriptors for CIP, ROX and SMX to characterize potential reactive sites, including frontier molecular orbitals (HOMO/LUMO), electrostatic potential (ESP) maps, and Fukui functions (see Text S6 for details).

**Fig. 5:**
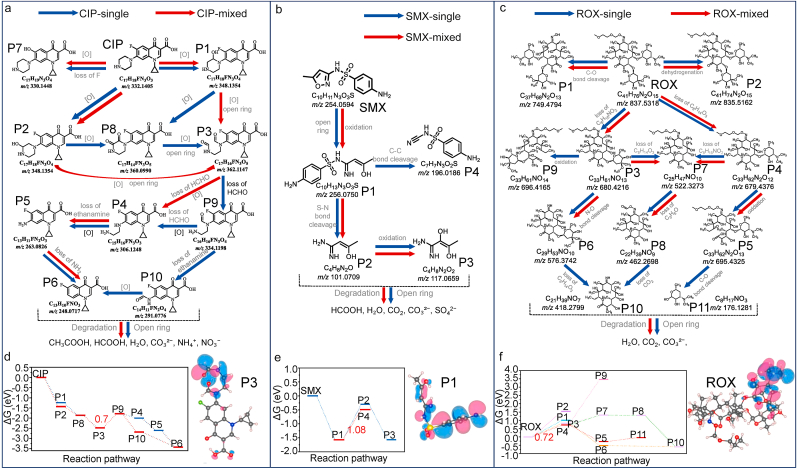
Proposed biodegradation pathways for (a) CIP, (b) SMX, and (c) ROX in the single- (blue arrows) and mixed-antibiotic (red arrows) groups. The energy barrier step diagrams between products of the reaction pathways for (d) CIP, (e) SMX, and (f) ROX groups, as well as the active electron distribution images of the starting material for the highest energy barrier steps.

Fig. 5a–f illustrated the proposed degradation pathways and the associated energy barrier analyses for the three antibiotics in the single- and mixed-antibiotic groups. As shown in Fig. 5a–d, the degradation of CIP in the single-antibiotic group was initiated by successive oxidative hydroxylation at distinct methylene carbons of the piperazine ring, yielding two isomeric intermediates P1 (C_17_H_18_FN_3_O_4_) and P2 (C_17_H_18_FN_3_O_4_) with an *m*/*z* of 348. Subsequently, P1 and P2 underwent oxidative dehydrogenation to form product P8 (*m*/*z* = 360). The piperazine ring of P8 was then subjected to further oxidative ring-opening to generate product P3 (*m*/*z* = 362). Subsequently, P3 underwent deformylation (loss of –CHO) to produce P9 (*m*/*z* = 334). Further deformylation of P9 yielded product P4 (*m*/*z* = 306), which was then converted to P5 (*m*/*z* = 263) via the removal of an ethylamine group. The amino group of P5 could be subsequently removed through oxidation to yield P6 (*m*/*z* = 248). Alternatively, the removal of an ethylamine group from P9 could produce P10 (*m*/*z* = 291), which was then converted to P6 through deformamidation. Additionally, the parent compound could undergo defluorination followed by oxidative hydroxylation to generate product P7 (*m*/*z* = 330). The products from these stages would undergo further degradation, ultimately leading to mineralization into inorganic substances (e.g., H_2_O, CO_2_).

In the mixed-antibiotic group, the initial degradation of CIP was consistent with that in the single group (yielding P1/P2), but the subsequent pathway was significantly simplified into three dominant routes. In two of the identified pathways, P1 and P2 were directly converted to P3 via oxidative ring-opening, which was then degraded to P6 via P4 and P5, bypassing intermediates P8, P9, and P10. In pathway 3, CIP was converted to P7 via defluorination.

For SMX in the single-antibiotic group (Fig. 5b), the first pathway involved an initial oxidative ring-opening to form P1. Subsequently, cleavage of the S−N sulfonamide bond released P2, which was further oxidized to P3. In the second pathway, intermediate P1 was converted to P4 via the oxidative cleavage of a C−C bond. The final products of both pathways were small inorganic molecules (H_2_O, CO_2_, CO_3_^2−^, H_2_SO_4_). However, the biodegradation of SMX in the mixed-antibiotic group followed only a single pathway (Fig. 5b), as pathway 2, which proceeds through intermediate P4, was not detected.

Seven degradation pathways were identified for ROX in the single-antibiotic group (Fig. 5c), involving multiple reaction types such as oxidative dehydrogenation, C−O bond cleavage, and ring-opening, all closely related to the functional groups in its molecular structure (e.g., amino, ester, and ether groups). In pathways 1 and 2, ROX was either converted to P1 (*m*/*z* = 749) via oxidative dehydrogenation or through the cleavage of the C−O bond in the 2-[(methoxyethoxy)-methyl]oxime substituent. Pathways 3–5 were all initiated by the cleavage of the C−O bond in the 5-(4-dimethylamino-tetrahydro-3-hydroxy-6-methyl-2-pyranoxy) substituent, leading to the loss of a C_8_H_15_NO_2_ moiety and the formation of product P3 (*m*/*z* = 680). P3 then branched into three sub-pathways: (i) cleavage of the C−O bond at the 3-(tetrahydro-5-hydroxy-4-methoxy-4,6-dimethyl-2-pyranoxy) position, releasing a C_8_H_14_O_3_ moiety to form P7 (*m*/*z* = 522); (ii) oxidative ring-opening of the lactone to yield P9 (*m*/*z* = 696); or (iii) cleavage of the C−N bond in the oxime group, releasing 2-[(methoxyethoxy)-methanol] to form P6 (*m*/*z* = 576).

In pathways 6 and 7, ROX was first converted to P4 (*m*/*z* = 679) through C−O bond cleavage at the 3-(tetrahydro-5-hydroxy-4-methoxy-4,6-dimethyl-2-pyranoxy) position, releasing a C_8_H_14_O_3_ moiety. P4 was then further degraded via two routes. The first route involved C−O bond cleavage at the 5-(4-dimethylamino-tetrahydro-3-hydroxy-6-methyl-2-pyranoxy) substituent, releasing a C_8_H_15_NO_2_ moiety to form P7 (*m*/*z* = 522). P7 was further degraded to P8 (*m*/*z* = 462) via the loss of a methyl ethyl ether group and subsequently to P10 (*m*/*z* = 418) through decarboxylation. The second route involved oxidative ring-opening of the lactone to produce P5 (*m*/*z* = 695), which was then cleaved to yield 3,4,6-trideoxy-3-(dimethylamino)-β-D-xylo-hexopyranose (P11; *m*/*z* = 176). In the mixed-antibiotic group, the biodegradation pathway of ROX was simplified to six routes, primarily characterized by the omission of certain pathways and steps. Specifically, pathway 4 was not detected, and the P10 step in pathway 3 and the P11 step in pathway 5 were absent.

A comparison of the degradation of the three antibiotics in the single- and mixed-antibiotic groups revealed that for a given antibiotic, the core degradation sites and dominant pathways remained consistent across both systems. The primary differences lay in the number and complexity of the pathways and the diversity of the intermediate products. The single-antibiotic groups exhibited more numerous and complex pathways, whereas in the mixed group, fewer products were detected, and the pathways were more direct, often showing “skip-step” reactions (Fig. S8). This is related to the intrinsic properties of the antibiotics and the energy requirements of the reactions. DFT calculations (Fig. 5d–f; see Texts S7–S9 and Figs. S5–S7 for details) indicated that under the competitive pressure and adjusted energy resource allocation in the mixed-antibiotic system, microorganisms preferentially selected degradation pathways with lower energy barriers. The environmental relevance of the identified transformation products was further evaluated using ECOSAR-based toxicity prediction, with detailed results provided in Text S10.

### 3.5 Metagenomics-based mechanistic interpretation

#### 3.5.1 Migration and transformation mechanisms in single-antibiotic groups

**Fig. 6:**
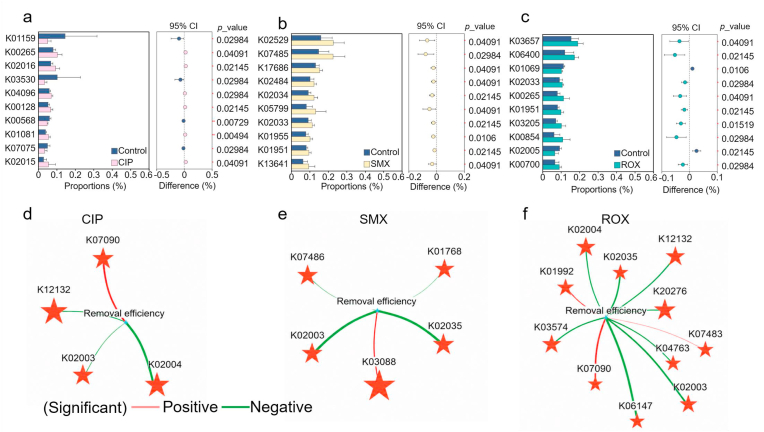
(a–c) Significantly different KOs in the (a) CIP, (b) SMX, and (c) ROX groups compared to the control, identified using the Wilcoxon rank-sum test. (d–f) KOs significantly correlated with antibiotic removal efficiency in the single-antibiotic groups. In panels (d–f), the size of nodes represents the abundance of species or functions; the thickness of lines indicates the strength of the correlation. CI, confidence interval.

**Fig. 7:**
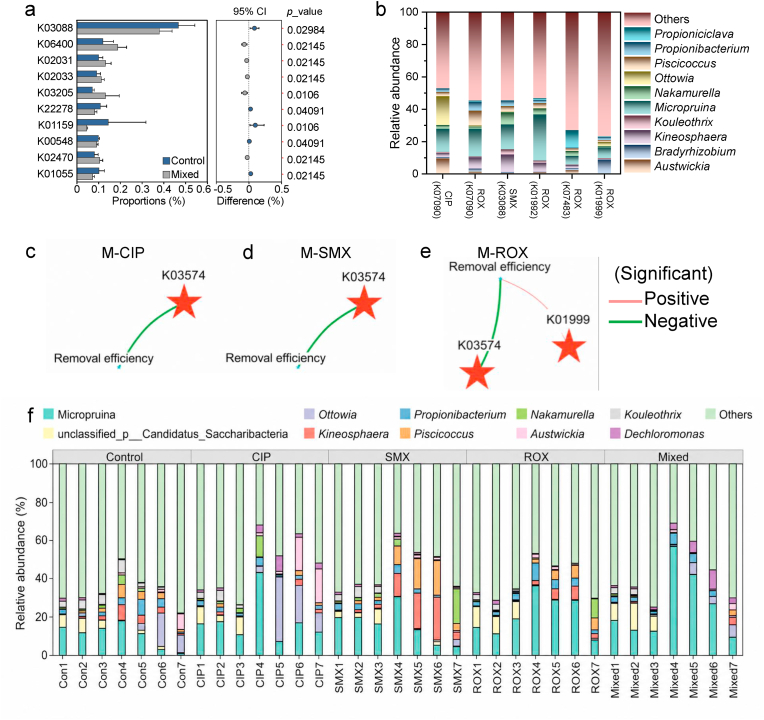
(a) Significantly different KOs in the mixed groups compared to the control, identified using the Wilcoxon rank-sum test. (b) Bar plot of species and functional contribution analysis at the genus level for KOs positively correlated with antibiotic removal. (c–e) KOs significantly correlated with antibiotic removal efficiency in the mixed-antibiotic groups (the size of nodes represents the abundance of species or functions; the thickness of lines indicates the strength of the correlation). (f) Genus-level community composition across groups and time points (1–7 represent days 15, 45, 75, 105, 135, 165, and 195, respectively).

To further explain these observed behaviors at the microbial and functional levels, metagenomics analyses were conducted. In the single-antibiotic groups, the migration and transformation of different antibiotics exhibited distinct microbial community adaptations with a strong dependence on molecular structure [30,31]. Metagenomic KEGG enrichment analysis revealed that in the CIP group (Fig. S9a), functional categories associated with cell-surface adhesion and membrane trafficking were enriched, including cell adhesion molecules (ko04514), adherens junctions (ko04520), tight junctions (ko04530), and endocytosis (ko04144). These KEGG pathway names are retained as annotation labels; the enrichment reflects bacterial homologs/orthologs and transport/adhesion-related functions mapped to these modules rather than eukaryotic junction or endocytosis processes per se. Collectively, these enrichments suggest strengthened cell–cell/cell–EPS interactions and potentially enhanced uptake-associated processes, which may increase biofilm- and EPS-mediated adsorption and sequestration of CIP. However, once challenged intracellularly, CIP primarily elicited a resistance network centered on drug efflux and DNA repair [32,33]. Key genes encoding ABC transporter systems, K02015 and K02016 (Fig. 6a), were significantly upregulated, and their core components, K02003 and K02004, showed significant negative correlations with CIP removal efficiency (Fig. 6d), suggesting that enhanced efflux may contribute to CIP persistence in the aqueous phase. Concurrently, the significant upregulation of the DNA processing protein K04096 (Fig. 6a) indicates active repair of CIP-induced damage. The limited biodegradation of CIP was statistically associated with *Ottowia* and *Micropruina* through the enrichment of the uncharacterized KO K07090, which showed a significant correlation with antibiotic removal performance (Fig. 7b and Text S11), consistent with previous reports identifying K07090 as a dominant KO under antibiotic stress [34].

The SMX group exhibited a concurrent biodegradation–resistance response. The low adsorption of SMX was associated with the suppression of biofilm-related pathways, such as quorum sensing (ko02024), under SMX stress (Fig. S9b) [35]. Previous studies have reported that K03088 is involved in SMX biodegradation [36]. In our study, under long-term SMX pressure, *Micropruina* and *Kineosphaera* became dominant genera (Fig. 7f), and the abundance of K03088 contributed by these microorganisms showed a significant positive correlation with the SMX removal efficiency (Fig. 6e), supporting a statistically robust but correlative species–function association (Text S11). Meanwhile, the SMX-exposed community activated resistance networks, including the ABC transporter system ko02010 (Fig. S9b), transcriptional reprogramming (K02529), and horizontal gene transfer (K07485) (Fig. 6b). Together, the coexistence of degradative potential and systemic resistance likely shaped the fate of SMX, characterized by relatively high biodegradability and low adsorption, yet persistent occurrence in the effluent.

For ROX, its migration and transformation displayed a persistence pattern governed by dominant biofilm/EPS-associated adsorption, enhanced efflux resistance, and constrained biodegradation. Metagenomic analysis showed that community remodeling under ROX stress led to *Micropruina* becoming a dominant genus (Fig. 7f). This genus contributed key genes, K01992 and K07090, which were positively correlated with ROX removal efficiency (Fig. 7b). Specifically, the ABC-2 type transport protein K01992 (Fig. 6f) may participate in ROX-related transport-associated processes, while the uncharacterized protein K07090 (Fig. 6f) likely mediates intracellular transformation. Nevertheless, this limited biodegradation potential appeared insufficient to offset the high intrinsic chemical stability of ROX (initial energy barrier of 0.72 eV), strengthened adsorption associated with activation of glycerolipid metabolism (ko00561; Fig. S9c), and the competitive advantage of a “survival-first” strategy, including enhanced ABC transporter-related transport responses involving K02033 and DNA damage repair (K03657) (Fig. 6c). Consequently, ROX exhibited substantial enrichment in sludge and persistence in the effluent.

#### 3.5.2 Migration and transformation mechanisms in the mixed-antibiotic group

Under combined stress from CIP, SMX, and ROX, the microbial community constructed an efficient efflux network by systemically upregulating the ABC transporter system ko02010, which was also significantly upregulated at the proteome level (Fig. S9d). Its enriched components, including K02031 (ddpD) and K02033 (ABC.PE.P) (Fig. 7a), indicate activation of an ABC transporter-related membrane transport module under mixed stress. Given that K02033 is annotated in KEGG as a peptide/nickel transport system permease protein, we interpret this signal conservatively as transport-associated adaptation rather than direct standalone evidence of multidrug antibiotic efflux. As shown in Fig. 7a, the Type IV secretion system protein VirD4 (K03205) may facilitate horizontal transfer of resistance genes, DNA gyrase gyrB (K02470) may reduce CIP efficacy through target modification [33], and the site-specific recombinase spoIVCA (K06400) may promote adaptive genomic recombination.

The emergence of *Zoogloea* as a dominant genus (Fig. 7f), together with enrichment of the cell cycle–Caulobacter pathway (ko04112; Fig. S9d), suggests intensified growth regulation and biofilm-related adaptation, consistent with increased EPS production that can provide additional adsorption sites for antibiotics [37]. Furthermore, metagenomic analysis indicated that antibiotic biodegradation potential was under complex regulation, involving both activation and inhibition. On one hand, the community enriched starch and sucrose metabolism (ko00500; Fig. S9d), and the significant enrichment of β-glucosidase (bglB, K05350) (Fig. S9f) suggests active polysaccharide hydrolysis to acquire carbon and energy [38]. This metabolic shift may support co-metabolic transformation of antibiotics, consistent with the upregulation of pathways involved in polycyclic aromatic hydrocarbon (ko00624) and furfural (ko00365) degradation (Fig. S9d). The emergence of *Dechloromonas* (a potential executor of aromatic co-metabolism) in the community composition profile (Fig. 7f), together with the identification of *Micropruina* (a major contributor to KOs positively correlated with the degradation of all three antibiotics) further supported the species–function linkage (Fig. 7b).

However, this biodegradation potential was attenuated by several constraints. A “survival-first” response pattern was suggested by the combined upregulation of transport- and repair-related functions, including 8-oxo-dGTPase K03574 (Fig. 7c–e), together with suppression of pathways related to cofactor synthesis (ko01240) and secondary metabolite synthesis (ko01110) (Fig. S9d), which may limit the activity of degradative enzymes and the supply of key coenzymes (e.g., NADH), thereby suppressing transformation capacity [33]. This interpretation is supported by multi-omics correlations with antibiotic removal efficiency rather than by direct causal validation. In summary, the migration and transformation of antibiotics under mixed-antibiotic stress reflect multiscale synergistic effects: although dominant genera and metabolic activation provided potential for transformation, the antibiotics’ intrinsic stability, differential interactions with EPS, and community-level resistance/repair and metabolic inhibition collectively shaped the ultimate outcome of partial degradation and widespread persistence.

## 4 Conclusion

This study systematically investigated the migration and transformation mechanisms of three antibiotics with moderate-to-high risk in biological wastewater treatment, suggesting that their divergent environmental fates are shaped by the macroscopic metabolic reconfiguration and microscopic molecular interactions within the microbial community. Specifically, during long-term operation, the antibiotic removal efficiency in the aqueous phase followed the order SMX > ROX ≈ CIP, while the adsorption affinity in the sludge phase was CIP > ROX > SMX. EPS characterization (PS/PN, FTIR, and 3D-EEM) consistently identified protein-like moieties as dominant sorption sites. Mechanistically, simulations suggest preferential binding to the protein fraction and support an interpretable co-adsorption behavior under mixed exposure, where CIP anchors via multipoint hydrogen bonding/electrostatic interactions and is associated with SMX stabilization within the same pocket through aromatic interactions, increasing co-retention potential. DFT-based reactivity descriptors and barrier analyses support the proposed pathways and rationalize simplified “skip-step” transformations in the mixed system.

Under long-term exposure to individual antibiotics, the microbial communities showed enrichment of bacterial genera significantly associated with antibiotic removal (e.g., *Micropruina*, *Ottowia*, *Kineosphaera*), coinciding with greater diversity of reaction products and more complex degradation pathways. In contrast, the combined stress from mixed-antibiotics was characterized by a “survival-first” strategy. Responses such as the activation of transporter-related stress responses, stress repair mechanisms, and metabolic inhibition were more prominent and were accompanied by lower antibiotic degradation rates in the mixed-antibiotic group compared to the single-antibiotic groups. Furthermore, microorganisms in the mixed group exhibited transformation patterns consistent with thermodynamically more favorable, “skip-step” pathways with lower energy barriers.

This study advances the understanding of the migration, transformation, and biodegradation mechanisms of antibiotics in activated sludge systems, providing a mechanistic basis for optimizing technologies for antibiotic removal in biological wastewater treatment.

## CRediT authorship contribution statement

**Bingqing Wang:** Formal analysis, Software, Writing – original draft, Writing – review & editing. **Zuxin Xu:** Conceptualization, Funding acquisition, Methodology, Resources. **Bin Dong:** Formal analysis, Supervision, Validation.

## Declaration of competing interest

The authors declare that they have no known competing financial interests or personal relationships that could have appeared to influence the work reported in this paper.
