# Supplementary material for: Migration and biotransformation mechanisms of risk-priority antibiotics in wastewater biotreatment: An integrated multi-omics and molecular dynamics perspective
Source: Eco Environ Health. 2026 Jun 25;5(3):100260. doi: 10.1016/j.eehl.2026.100260 (PMC13382025; doi:10.1016/j.eehl.2026.100260)
Supplement: Multimedia component 1 [file mmc1.docx]

Supporting Information

**Migration and biotransformation mechanisms of risk-priority antibiotics in wastewater biotreatment: An integrated multi-omics and molecular dynamics perspective**

BingQing Wang^a,b^, Zuxin Xu^a,b,*^ Bin Dong^a,b,*^

^a^ State Key Laboratory of Pollution Control and Resource Reuse, College of Environmental Science and Engineering, Tongji University, Shanghai 200092, China

^b^ Ministry of Education Key Laboratory of Yangtze River Water Environment, Tongji University, Shanghai 200092, China

-------------------------------------

*Corresponding authors.

E-mails: xzx@tongji.edu.cn (Z. Xu); dongbin@tongji.edu.cn (B. Dong)

This Supplementary Information (SI) provides detailed methodological descriptions, additional figures, and analyses supporting the main text. Texts S1–S6 cover reactor operation, analytical methods, EPS characterization, molecular simulations, and metagenomics/metaproteomics analyses. Texts S7–S9 present detailed degradation pathways and energy barrier analyses for ciprofloxacin (CIP), sulfamethoxazole (SMX), and roxithromycin (ROX), respectively. Texts S10–S13 include ecotoxicity prediction of transformation products, correlation analysis between KEGG Orthology (KO) abundance and antibiotic removal efficiency, convergence checks and binding free energy estimation in molecular dynamics simulations, and discussion of the scope and limitations of pathway and energy inference with recommended validations. Readers are advised to follow the sequence for consistency with the main text.

**Text S1 Detailed reactor operation and physicochemical/analytical methods**

To simulate biological municipal wastewater treatment, five sequencing batch reactors (SBRs; working volume 4.0 L each) were established, including a control reactor, three single-antibiotic reactors (CIP, SMX, and ROX), and a mixed-antibiotic reactor. All reactors were configured for simultaneous carbon removal and biological nitrogen and phosphorus removal, and the effluent consistently met the Chinese Grade 1B discharge standard (GB 18918−2002), supporting their representative nature for municipal biological wastewater treatment.

All reactors were operated under identical conditions (Table S1). Two 12-h cycles were performed per day. Each cycle consisted of 2.0 L influent feeding and 2.0 L effluent withdrawal, maintaining a constant working volume of 4.0 L (daily treated volume 4.0 L; HRT ≈ 1 d). Solids retention time (SRT) was maintained at 20 d by daily wasting a fixed amount of sludge. Reactors were operated at room temperature without additional temperature control. No aeration was provided during the mixing stage, and dissolved oxygen (DO) remained at 0.16–0.24 mg/L. Aeration was supplied at approximately 1.0 L/min and controlled using a gas flow meter. No external pH adjustment was applied. Antibiotics were introduced after a 20 d acclimation period by dosing with the influent; the target concentration was 1.0 mg/L for each compound in the single-antibiotic reactors, and for the mixed reactor, CIP, SMX, and ROX were simultaneously dosed at an equal proportion (1:1:1), i.e., 1.0 mg/L for each antibiotic (total 3.0 mg/L). This dosing level was selected as a mechanism-oriented, class-representative stress condition. The composition of synthetic wastewater is provided in Table S2.

FTIR spectra were acquired using a Thermo Fisher Scientific Nicolet iS20 with a DTGS KBr detector and KBr beam splitter. Spectra were collected over 400–4000 cm⁻¹ with 32 scans for both sample and background at a resolution of 4 cm⁻¹ (sampling gain 1.0; mirror velocity 0.4747; aperture 150). Antibiotics in influent and effluent (CIP, SMX, and ROX) were quantified by SPE–LC–MS/MS following the Shandong provincial standard DB37/T 3738–2019 (*Water quality—Determination of antibiotics of sulfonamides, quinolones and macrolides by solid phase extraction—Liquid chromatography triple quadrupole mass spectrometry*). Sludge-phase antibiotics were extracted and cleaned up according to the agricultural industry standard NY/T 3787–2020 (*Simultaneous determination of tetracyclines, fluoroquinolones, sulfonamides, macrolides and chloramphenicols in soil by HPLC method*), including freeze-drying of sludge samples, SPE clean-up, and instrumental determination. Transformation products were screened using UPLC–QTOF–MS on an Agilent 1290 UPLC coupled to an Agilent 6550 Q-TOF. Chromatographic conditions were: mobile phase A, 0.1% formic acid in water; mobile phase B, acetonitrile; flow rate 0.3 mL/min; injection volume 5 μL; column, Waters BEH C18 (2.1 mm × 100 mm, 1.7 μm). MS conditions included a full-scan range of 50–1000 *m/z*; sheath gas temperature 350 ℃ and sheath gas flow 12 L/min; capillary voltage 4000 V in ESI+ and 3200 V in ESI− mode.

**Antibiotic fate analysis and apparent mass balance calculation**

The fate of antibiotics in activated sludge was evaluated using a mass-balance approach adapted from the phase-partitioning strategy reported by Wang et al. (2024). During stable operation, influent, effluent, and mixed liquor samples were collected. Immediately after feeding and complete mixing, mixed liquor samples were collected to determine the initial reactor concentration at the beginning of the cycle (*C*_in_). Mixed liquor samples were centrifuged to separate the sludge pellet from the reactor liquid phase, and EPS was subsequently extracted from the pellet to obtain an EPS extract. Antibiotic concentrations were quantified in the reactor aqueous phase (*C*_aq_, mg/L), in the EPS extract as EPS-associated adsorption (C_ads-EPS_, normalized to reactor volume, mg L^−1^), and in the residual sludge after EPS extraction as sludge-associated adsorption (C_slu_, normalized to dry solids, mg/kg dry sludge). This operational definition enabled the separation of EPS adsorption from adsorption to the bulk sludge matrix. Based on these measurements, the net biotransformation in the sludge system was calculated by difference:

$$C_{\mathrm{bio}\text{-}\mathrm{slu}}=C_{\mathrm{in}}-\left( C_{\mathrm{aq}}+C_{\mathrm{ads}\text{-}\mathrm{EPS}}+C_{\mathrm{slu}}\cdot X_{\mathrm{MLSS}} \right)$$

where *C*_bio-slu_ is the apparent net biotransformed amount (mg/L), *C*_in_ is the initial reactor concentration measured immediately after feeding and complete mixing at the beginning of the cycle (mg/L), rather than the external feed concentration, *C*_aq_ is the residual antibiotic concentration in the effluent aqueous phase (mg/L), *C*_ads-EPS_ is the EPS-associated adsorption normalized to reactor volume (mg/L), *C*_slu_ is the sludge-associated antibiotic concentration after EPS extraction (mg/kg dry sludge), and *X*_MLSS_ is the mixed liquor suspended solids concentration (kg/L). To ensure dimensional consistency, all fractions were converted to volume-normalized concentrations (mg/L) before calculation. Because *C*_in_ was measured after feeding and complete mixing, the effect of the 50% volume exchange was already incorporated into the measured initial concentration. Therefore, no additional exchange-ratio correction was introduced. This framework partitions antibiotic removal into aqueous residuals, EPS adsorption, sludge adsorption, and the remaining fraction attributed to apparent net biotransformation.

**Text S2** **Computational methods for degradation pathway analysis**

Spin-polarized density functional theory (DFT) calculations were performed using the Vienna *Ab initio* Simulation Package (VASP) [[1](#_ENREF_1),[2](#_ENREF_2)] employing the projected augmented wave (PAW) pseudopotentials [[3](#_ENREF_3)]. The exchange–correlation interactions were described using the generalized gradient approximation (GGA) with the Perdew–Burke–Ernzerhof (PBE) functional [[4](#_ENREF_4),[5](#_ENREF_5)]. A plane-wave kinetic energy cutoff of 450 eV was applied throughout all calculations. All molecular models were constructed in a cubic unit cell with dimensions of 21 Å × 21 Å × 21 Å. A vacuum region of at least 15 Å was introduced in all directions to eliminate spurious interactions between periodic images. Brillouin zone sampling was carried out using the Monkhorst–Pack scheme [[6](#_ENREF_6)] with a k-point spacing of 0.05 Å^-1^ corresponding to a 1 × 1 × 1 k-point mesh. Geometry optimizations were conducted using the quasi-Newton limited-memory Broyden–Fletcher–Goldfarb–Shanno (l-BFGS) algorithm until the total energy and maximum residual force on each degree of freedom were converged to within 1.0 × 10^-5^ eV and 0.01 eV/Å, respectively. The SMD implicit solvation model was used to simulate the real solution.

To construct the free energy profile of the degradation pathways, Gibbs free energies of all stationary points were evaluated based on zero-point energy (ZPE)-corrected DFT total energies, which were taken as the ground-state energy at 0 K. The Gibbs free energy change (ΔG) was calculated according to the following equation:

$$\Delta G=\Delta H-T\Delta S= \Delta E_{DFT}+ {\Delta E}_{ZPE}+ \int_{0}^{T} {\Delta C}_{V}dT-T\Delta S$$

where ΔE_DFT_ (eV) represents the electronic energy difference obtained from DFT optimizations (including solvation effects), ΔE_ZPE_ (eV) denotes the zero-point vibrational energy correction, ΔC_V_ (eV K^−1^) is the difference in heat capacity at constant volume, T (K) is the absolute temperature (298.15 K), ΔS (eV K^−1^) is the entropy change, and ΔG (eV) is the Gibbs free energy change. Thermodynamic corrections for gas-phase species were derived using standard statistical thermodynamics based on tabulated data at standard conditions obtained from the NIST database. It should be noted that the DFT-derived energy barriers reflect the intrinsic chemical feasibility of transformation pathways, rather than the actual enzymatic kinetics in the biological system. Therefore, the calculated energy profiles are used to compare the relative favorability of different transformation routes, rather than to directly represent reaction rates in activated sludge.

**Text S3 Computational methods for protein docking**

**Topology file preparation**

The structures of small molecules, including antibiotics and the Temple–Nightingale–Birmingham (TNB) model compound representing humic acid [[7](#_ENREF_7)], were optimized using the ORCA program at the B3LYP-D3(BJ)/6-311G(d,p) level [[8](#_ENREF_8)], followed by frequency calculations to confirm that the optimized structures correspond to local minima on the potential energy surface. Based on the optimized geometries, RESP charges were calculated using the Multiwfn program [[9](#_ENREF_9)]. Subsequently, GAFF force field topologies for the antibiotic and TNB molecules were generated using Sobtop [[10](#_ENREF_10)]. The Amber14SB force field topology for the protein was generated using the pdb2gmx tool in GROMACS [[11](#_ENREF_11)]. A decasaccharide composed of ten glucose units linked via α-1,4-glycosidic bonds was employed as the model polysaccharide, while humic acid was represented using the widely adopted Temple–Nightingale–Birmingham (TNB) model [[7](#_ENREF_7)].

**Molecular dynamics simulation**

Bovine serum albumin (BSA) was employed as a representative protein component of extracellular polymeric substances. The polysaccharide and humic acid models described above were incorporated into the EPS simulation system. To mimic the spontaneous self-assembly of EPS constituents, the initial simulation system containing protein, polysaccharide, humic acid, and antibiotic molecules was constructed using Packmol, with the mass ratio of protein : polysaccharide : humic acid set to 5 : 3 : 1, corresponding to a molecular ratio of BSA : polysaccharide : TNB = 1 : 25 : 18. The resulting complex was subsequently solvated in a cubic box with dimensions of 12 nm × 12 nm × 12 nm. This composite EPS system was intended as a representative minimal model to capture the dominant interactions between antibiotics and major EPS-like components, rather than to reproduce the full structural heterogeneity of natural sludge EPS. In contrast, for mechanistic investigations focusing on specific protein–antibiotic interactions, the outer membrane protein OmpA was considered. The OmpA structure used in this study was obtained from the AlphaFold Protein Structure Database (AF-Q05146-F1-v6). Only the soluble domain (residues 77–193) was retained for simulation, while the transmembrane region was excluded. Complexes between the retained OmpA domain and the three antibiotics (ciprofloxacin, roxithromycin, and sulfamethoxazole) were then used as the initial models for molecular dynamics simulations. The protein-antibiotics complex was placed at the center of a cubic box with a minimum distance of 1.5 nm from the box edge, which was subsequently solvated with TIP3P water molecules. Appropriate numbers of Na^+^ and Cl^-^ ions were added to neutralize the system.

Energy minimization was initially carried out using the steepest descent algorithm with a maximum of 5000 steps and an energy convergence criterion of 1000 kJ mol⁻¹ nm⁻¹. Non-bonded interactions were treated using the Verlet cutoff scheme, with both van der Waals and short-range Coulomb cutoff distances set to 0.9 nm, while long-range electrostatic interactions were calculated using the particle mesh Ewald (PME) method. For the BSA-based EPS self-assembly system, no position restraints were applied throughout the simulation process, allowing the protein, polysaccharide, humic acid, and antibiotic molecules to spontaneously reorganize and self-assemble under thermal motion. In contrast, for the protein–antibiotic complex systems involving OmpA, position restraints were applied to both the protein and the bound antibiotic during the energy minimization and equilibration stages to preserve the structural integrity of the complex and prevent premature dissociation during solvent relaxation. Following minimization, an NVT equilibration was performed for 125 ps with a 1 fs time step. The system temperature was maintained at 298.15 K using the velocity-rescaling (v-rescale) thermostat with a coupling constant of 1.0 ps, applied separately to the protein and solvent. Initial velocities were generated according to the target temperature, and non-bonded interaction parameters were kept consistent with those used during energy minimization. Position restraints on the protein and antibiotic were retained throughout this equilibration phase. Subsequently, a production molecular dynamics simulation was conducted under NPT conditions for 100 ns for the BSA system and 200 ns for the OmpA system with a 2 fs time step, after removing all position restraints. The temperature was maintained at 298.15 K, and the pressure was isotropically controlled at 1 bar using the Parrinello-Rahman barostat with a coupling constant of 5.0 ps. All non-bonded interaction parameters, including the cutoff scheme and cutoff distances, remained unchanged. Linear momentum was periodically removed, and angular momentum correction was applied to the solute to ensure numerical stability and physical accuracy of the simulations.

**Text S4 Detailed metagenomic and metaproteomic bioinformatic analysis**

Metagenomic libraries were sequenced on an Illumina NovaSeq^TM^ X Plus platform using paired-end 150 bp reads, generating approximately 6–10 Gb of clean data per sample. Taxonomic and functional annotations were performed against the NCBI NR database (version 2020-06-04) and the KEGG database (release 94.2). Metaproteomic analyses were conducted in data-independent acquisition (DIA) mode using an Orbitrap Astral mass spectrometer, and protein identifications were accepted at a false discovery rate (FDR) of ≤1%.

Raw paired-end metagenomic reads were quality-filtered using fastp v0.20.0 to remove adapters and low-quality sequences. Clean reads were assembled de novo with MEGAHIT v1.1.2, and contigs ≥300 bp were retained. Open reading frames (ORFs) were predicted using Prodigal v2.6.3, and genes ≥100 bp were used for downstream analyses. A non-redundant gene catalog was constructed using CD-HIT v4.7 (90% sequence identity and coverage), with the longest sequence selected as the representative gene for each cluster.

Clean reads were mapped to the non-redundant gene catalog using SOAPaligner v2.21 (≥95% identity), and gene abundance was normalized as RPKM (reads per kilobase per million mapped reads). Taxonomic annotation was performed against the NCBI NR database using DIAMOND v2.0.13 (e-value ≤ 1×10^-5^), and functional annotation was primarily based on the KEGG database (release 94.2) to obtain KO assignments and pathway information.

Because this study was based on one reactor per treatment condition, samples collected at different time points from the same reactor were not treated as independent biological replicates but as time-series observations used to characterize system dynamics under long-term antibiotic stress. For statistical analyses, raw *p* values were adjusted using FDR method implemented in the Majorbio platform, and significance criteria were reported explicitly for each analysis. This clarification was added to avoid over-interpreting temporal samples as independent biological replicates.

In the Majorbio platform, metagenomic differential analyses were based on a two-group comparison strategy, primarily between the control group and each treatment group. For each two-group comparison, differential KO and species abundances were tested using the Wilcoxon rank-sum test (two-sided). Raw p values were adjusted using FDR correction for multiple comparisons, and FDR-adjusted *p* < 0.05 was used as the significance threshold. Functional abundance was normalized as RPKM. KEGG enrichment analysis was conducted using the reporter_score (GRSA-based reporter_score) method. Correlation analysis between functional abundance (KO) and environmental variables was performed using Spearman correlation, with |ρ| ≥ 0.5 and FDR-adjusted *p* < 0.05 regarded as significant.

Metaproteomic data were analyzed using the Majorbio standardized pipeline. Protein identifications were accepted at Q-value < 0.01, and protein abundance was calculated based on high-confidence peptide signals. Identified proteins were mapped to KEGG to obtain KO and pathway annotations, enabling integrative comparisons with metagenomic functional profiles.

Differentially expressed proteins were defined using the criteria |log_2_ fold change| > 1 and FDR-adjusted *p* < 0.05, with identification confidence controlled at Q-value < 0.01. Group-wise comparisons of taxonomic and functional profiles were performed using statistical modules implemented in the Majorbio platform, with non-parametric tests applied where appropriate. Correlation analyses were conducted between normalized multi-omics features and reactor-level phenotypes (e.g., antibiotic removal efficiency and phase partitioning), with significance defined at FDR-adjusted *p* < 0.05.

Multiple-testing correction was applied during differential protein analysis. Raw *p* values were adjusted using FDR approach implemented in the Majorbio platform, and the resulting FDR-adjusted p values were reported as “corrected_pvalue” in the output tables. Proteins were considered significantly differentially expressed only when both |log_2_FC| > 1 and FDR-adjusted *p* < 0.05 were satisfied.

KEGG functional enrichment analysis was performed on the Majorbio Cloud platform to evaluate pathway-level functional shifts across groups. Briefly, KO profiles derived from metagenomic annotation (RPKM-normalized KO abundance table) were subjected to two-group comparison (e.g., each antibiotic group versus the control) to obtain differentially changed KOs. The platform then mapped these differential KO signals to KEGG pathways and calculated a pathway-level reporter_score (GRSA-based reporter_score algorithm) for each pathway, which quantitatively reflects the enrichment tendency/intensity at the pathway level. Pathways were ranked and interpreted based on their reporter_score outputs, and the enriched pathways were visualized in Fig. S9.

For sludge metaproteomics, proteins were extracted using a phenol-based method. Briefly, sludge samples were suspended in BPP buffer, homogenized, extracted with Tris-saturated phenol, and precipitated using ammonium acetate/methanol. The pellet was washed with pre-cooled acetone and dissolved in a lysis buffer containing 8 M urea and 1% SDS. After reduction and alkylation, proteins were digested with trypsin prior to DIA analysis. DIA raw files were processed using Spectronaut (version 19) against the non-redundant metagenome-derived protein catalog. Search parameters included trypsin/P specificity, a maximum of 2 missed cleavages, carbamidomethylation of cysteines as a fixed modification, oxidation of methionines and protein N-terminal acetylation as variable modifications, and FDR control at ≤1% at both the peptide and protein levels.

**Text S5 OmpA selected as a molecular model for studying antibiotic interactions at the sludge EPS interface**

Proteomics results were compared between the mixed-antibiotic group and the control to identify proteins significantly upregulated under antibiotic exposure as potential candidates. Screening criteria were set as an adjusted *p* value < 0.05 and a |log_2_ fold change| (FC, treatment/control) > 1 to ensure statistical significance and exposure relevance. Notably, some candidates were undetected in the control but were clearly expressed in the treatment group, a common phenomenon in high-throughput proteomics that typically reflects inducible responses to environmental stressors or xenobiotics rather than experimental noise.

Among the differential proteins, the OmpA family outer membrane protein (Outer membrane protein A, OmpA) exhibited significant upregulation under antibiotic exposure and aligned well with our mechanistic aims. OmpA is widely distributed in Gram-negative bacteria and is located at the cell–environment interface, where it can directly participate in interactions between extracellular molecules and microbial surfaces. Previous studies have also suggested that OmpA contributes to outer membrane integrity and interfacial interactions.

Importantly, OmpA has well-resolved three-dimensional structures, and its surface-exposed polar and hydrophobic residues provide plausible binding sites for small-molecule ligands, making it a tractable model for docking and MD simulations. Based on (i) significant differential expression in proteomics, (ii) availability of reliable 3D structural information, and (iii) biological plausibility as an interface-associated protein relevant to EPS–antibiotic interactions, OmpA was selected as the representative protein for molecular-scale analyses. Although outer membrane proteins are not classically defined as secreted EPS constituents, in activated sludge systems many cell-surface and membrane-associated proteins can become embedded in or tightly coupled to EPS networks via physical entanglement, electrostatic interactions and co-deposition processes, thereby contributing to antibiotic sorption and interfacial interactions. Accordingly, we used OmpA as a representative EPS-coupled interface protein model to probe protein site–mediated adsorption mechanisms in realistic sludge matrices.

**Text S6 Predicting reactive regions of antibiotic molecules via frontier orbital and fukui function analyses**

These quantum-chemical descriptors can identify molecular regions prone to electron transfer or radical attack without requiring explicit product information, thereby providing testable mechanistic clues for subsequent pathway interpretation. As shown in Fig. S2, the HOMO of CIP is mainly distributed on the fluoroquinolone core and the piperazine ring (−5.79 eV), whereas its LUMO is more concentrated on the carboxyl group (−1.35 eV). The HOMO–LUMO gap (4.44 eV) suggests relatively high electronic reactivity with both oxidative and reductive possibilities; moreover, prominent Fukui f⁻ regions are located around the piperazine ring, indicating this moiety as a preferential site for electrophilic attack. In comparison, ROX exhibits a larger HOMO–LUMO gap (6.13 eV), suggesting higher intrinsic stability and implying that reactive sites may be more localized on functional groups such as the glycosyl side chains. For SMX, frontier orbitals are mainly distributed on the sulfonamide and isoxazole rings, with a gap of 5.23 eV; the Fukui function also highlights stronger reactivity around the isoxazole ring and adjacent atoms, suggesting this region as a likely transformation hotspot. Notably, the theoretically predicted reactive sites are highly consistent with the experimentally proposed degradation pathways described below.

**Text S7 Ciprofloxacin biodegradation pathways and energy barriers**

**First degradation step (three possibilities):**
(1) Ciprofloxacin (sur) → P7: Hydroxyl substitution of the fluorine atom on the phenyl ring. The free energy change is +0.97 eV, indicating this reaction is thermodynamically unfavorable. (End of Pathway 5)
(2) Ciprofloxacin (sur) → P1: Hydroxyl substitution of a hydrogen atom at the α-carbon of the piperazine ring. The free energy change is -1.24 eV, indicating this reaction is highly favorable.
(3) Ciprofloxacin (sur) → P2: Hydroxyl substitution of a hydrogen atom at the β-carbon of the piperazine ring. The free energy change is -1.43 eV, indicating this reaction is highly favorable.

**Second degradation step (two possibilities):**
(1) P1 → P8: Oxidation of the hydroxyl and hydrogen atoms on the carbon of the piperazine ring. The free energy change is -0.62 eV, indicating a favorable reaction.
(2) P2 → P8: Oxidation of the hydroxyl and hydrogen atoms on the carbon of the piperazine ring. The free energy change is -0.43 eV, indicating a moderately favorable reaction.

**Third degradation step (one possibility):**
(1) P8 → P3: Oxidative cleavage adjacent to the carbonyl group to form a diamide. The free energy change is -0.62 eV, indicating a favorable reaction.

**Fourth degradation step (one possibility):**
(1) P3 → P9: Deformylation of one amide group in the diamide structure to form an amine. The free energy change is +0.70 eV, making this step unfavorable.

**Fifth degradation step (two possibilities):**
(1) P9 → P4: Deformylation of the other amide group to form an ethylenediamine structure. The free energy change is -0.22 eV, indicating a feasible reaction.
(2) P9 → P10: Loss of an ethylamine group to form a monoamide structure. The free energy change is -0.90 eV, indicating a favorable reaction.

**Sixth degradation step (two possibilities):**
(1) P4 → P5: Loss of an ethylamine group. The free energy change is -0.60 eV, indicating a favorable reaction.
(2) P10 → P6: Loss of the formamide group attached to the phenyl ring. The free energy change is -0.74 eV, indicating a favorable reaction. (End of Pathways 3 and 4)

**Seventh degradation step (one possibility):**
(1) P5 → P6: Loss of the amino group on the phenyl ring. The free energy change is +0.14 eV, making this step slightly unfavorable. (End of Pathways 1 and 2)

Therefore, the five degradation pathways for ciprofloxacin are illustrated in Figure S5. Among them, Pathway 3 (sur→P2→P8→P3→P9→P10→P6) is the most favorable. The P3→P9 step represents the highest energy barrier along this pathway (0.70 eV). Figure 5d shows the active electron distribution in P3, revealing that the electron density is concentrated on the distal amide bond (0.54 e), suggesting that this bond is more susceptible to reaction in subsequent degradation steps.

The designation of Pathway 3 as the most favorable pathway is based on the overall energetic profile of the reaction network rather than the barrier of an individual step.

**Text S8 Sulfamethoxazole biodegradation pathways and energy barriers**

**First degradation step (one possibility):**
(1) Sulfamethoxazole (sur) → P1: Oxidative cleavage of the N-O bond, leading to ring-opening. The free energy change is -1.57 eV, indicating this step is highly favorable.

**Second degradation step (two possibilities):**
(1) P1 → P2: Cleavage of the S-N bond, releasing *p*-aminobenzenesulfonic acid. The free energy change is +1.27 eV, making this reaction highly unfavorable.
(2) P1 → P4: Cleavage of a C-C bond. The free energy change is +1.08 eV, indicating it is unfavorable.

**Third degradation step (one possibility):**
(1) P2 → P3: Oxidation to form a vicinal diol. The free energy change is -1.27 eV, indicating a favorable process.

Therefore, the two degradation pathways for sulfamethoxazole are shown in Figure S6. Of the two, Pathway 2 (sur→P1→P4) is more favorable. The most difficult step is the conversion of P1 to P4, with an energy barrier of 1.08 eV. Thus, activation of the ring-opened SMX molecule itself could significantly lower the energy barrier for this step. Figure 5e calculates the active electron distribution in the ring-opened intermediate P1. It shows that, apart from the phenyl ring, the active electron density is dispersed, with only a small amount (0.11 e) concentrated around the reactive side chain. This suggests that introducing additional activating groups to the side chain could concentrate the active electron density and thereby enhance degradation performance.

**Text S9** **Roxithromycin biodegradation pathways and energy barriers**

**First degradation step (four possibilities):**

(1) Roxithromycin (sur) → P1: Elimination of the N-linked oxime ether group. The free energy change is +1.00 eV, making this reaction unfavorable. (End of Pathway 5)

(2) Roxithromycin (sur) → P2: Dehydrogenation and oxidation of a hydroxyl group to a carbonyl group. The free energy change is +1.54 eV, making this reaction highly unfavorable. (End of Pathway 6)

(3) Roxithromycin (sur) → P3: Loss of the C_8_H_15_NO_2_ moiety. The free energy change is +0.82 eV, indicating it is unfavorable.

(4) Roxithromycin (sur) → P4: Loss of the C_8_H_14_O_3_ moiety. The free energy change is +0.72 eV, indicating it is unfavorable.
**Second degradation step (five possibilities):**

(1) P3 → P6: Cleavage of the N-O bond, with the loss of a C_4_H_10_O_3_ moiety. The free energy change is -1.34 eV, indicating a favorable reaction.

(2) P3 → P7: Loss of the C₈H₁₄O₃ moiety. The free energy change is +0.49 eV, indicating it is unfavorable.

(3) P3 → P9: Oxidative cleavage of the vicinal diol structure. The free energy change is +2.56 eV, making this reaction extremely unfavorable. (End of Pathway 7)

(4) P4 → P7: Loss of the C₈H₁₅NO₂ moiety. The free energy change is +0.59 eV, indicating it is unfavorable.

(5) P4 → P5: Oxidative cleavage of the lactone bond. The free energy change is -1.00 eV, indicating a favorable reaction.

**Third degradation step (three possibilities):**

(1) P7 → P8: Oxidative elimination of a C₃H₈O moiety. The free energy change is -0.01 eV, indicating a feasible reaction.

(2) P5 → P11: Cleavage of a C-O bond to yield a C₈H₁₅NO₃ small molecule. The free energy change is +0.24 eV, indicating a feasible reaction. (End of Pathway 1)

(3) P6 → P10: Loss of the C_8_H_14_O_3_ moiety. The free energy change is -0.07 eV, indicating a feasible reaction. (End of Pathway 4)

**Fourth degradation step (one possibility):**

(1) P8 → P10: Cleavage of the N-O bond with the release of carbon dioxide. The free energy change is -1.90 eV, indicating a highly favorable reaction. (End of Pathways 2 and 3)

Therefore, the seven degradation pathways for roxithromycin are illustrated in Figure S7. Among them, Pathway 1 (sur→P4→P5→P11) is the most favorable. The most difficult step is the initial conversion of sur to P4, with an energy barrier of 0.72 eV. Thus, activation of the roxithromycin molecule itself could significantly lower the energy barrier for this rate-determining step. Figure 5f calculates the active electron distribution in roxithromycin, revealing that the electron density is concentrated on the C_8_H_15_NO_2_ moiety (0.72 eV). This suggests that further activation of this moiety could enhance the degradation performance.

**Text S10 Ecotoxicity prediction of transformation products based on ECOSAR**

To evaluate the environmental risks associated with the identified transformation products, ecotoxicity was predicted using the ECOSAR model. The predicted toxicity distributions for different antibiotic systems are shown in Fig. S10. Overall, most transformation products were distributed within low to moderate toxicity ranges (green to blue zones), indicating that the dominant transformation pathways do not lead to systematic increases in ecotoxicological risk. In contrast, only a limited number of intermediates fell into higher toxicity regions (orange to red zones), and these cases were not consistently observed across different antibiotic systems.

Notably, although certain transformation products exhibited slightly higher predicted toxicity than their parent compounds, these increases were scattered and limited in magnitude, without forming a consistent pattern of risk amplification. This suggests that the transformation processes identified in this study are more likely to result in products with comparable or only marginally increased ecological risks, rather than generating substantially more hazardous compounds. Taken together, these results indicate that the major transformation pathways identified in this study are unlikely to lead to significant accumulation of highly toxic intermediates.

**Text S11 Correlation analysis between KO abundance and antibiotic removal efficiency**

To further evaluate the association between microbial functional potential and antibiotic removal performance, a correlation network analysis was conducted using the Majorbio cloud platform. Spearman’s rank correlation analysis was applied to assess relationships between KO abundance and environmental variables (antibiotic removal efficiency), with a correlation coefficient threshold of |ρ| ≥ 0.5 and a significance level of FDR-adjusted *p* < 0.05.

The analysis revealed that several KOs, including K03088 in the SMX group and K07090 in the CIP and ROX groups, exhibited significant positive correlations with antibiotic removal efficiency. To further contextualize these associations, a species–function contribution analysis was performed for the significantly correlated KOs, identifying the dominant contributing genera (e.g., *Micropruina*, *Kineosphaera*, and *Ottowia*).

The consistent enrichment of specific genera, the elevated abundance of correlated KOs, and their statistically significant relationships with antibiotic removal efficiency collectively support a robust association between microbial community structure and functional potential. Nevertheless, these results represent correlation-based evidence, and direct causal validation through transcriptomic or in vitro enzymatic assays remains beyond the scope of the present study.

**Text S12 Convergence checks and binding free energy estimation in MD simulations**

To ensure the reliability of MD-derived interaction patterns, convergence and stability were evaluated using multiple metrics. For the OmpA–antibiotic systems, the protein backbone RMSD, ligand RMSD (relative to the protein), and minimum protein–ligand distance were monitored and showed stabilization after ~100 ns, supporting the use of the 100–200 ns trajectories for interaction statistics and representative conformations (Fig. S3).

For the composite EPS simulations, antibiotics interacted with three representative EPS components (protein, polysaccharide, and a humic-acid proxy), enabling component-resolved comparison. Binding free energy/energy trends reported in Fig. 3e were derived consistently from the equilibrated trajectories together with distance-based analyses, allowing quantitative comparison of relative binding strengths among EPS components (protein vs. polysaccharide vs. humic acid).

**Text S13 Scope and limitations of pathway/energy inference and recommended validations**

The biodegradation pathways presented in this study are *proposed* based on (i) intermediate products detected by TOF-MS and (ii) DFT-derived reactivity descriptors and energy barrier/free-energy profiles (Fig. 5 and Text S2). These results provide a mechanistic rationale for why mixed-antibiotic exposure may favor simplified routes (“skip-step” patterns) from a thermodynamic/kinetic perspective; however, they do not constitute direct enzymatic proof.

In addition, the inference of metabolic reprogramming and energy allocation under mixed stress (e.g., downregulation of cofactor synthesis modules such as ko01240) is currently supported by multi-omics pathway-level signals, while direct measurements of intracellular energy state (ATP/ADP ratios), redox cofactors (e.g., NADH/NAD^+^), or metabolomics profiling were not conducted. These measurements are recommended as future work to further validate the link between energy metabolism and degradation capacity. This interpretation of ko01240 downregulation as a reduced degradation capacity is based on pathway-level inference, supported by multi-omics evidence (metagenomic and metaproteomic data), rather than direct ATP or NADH measurements.


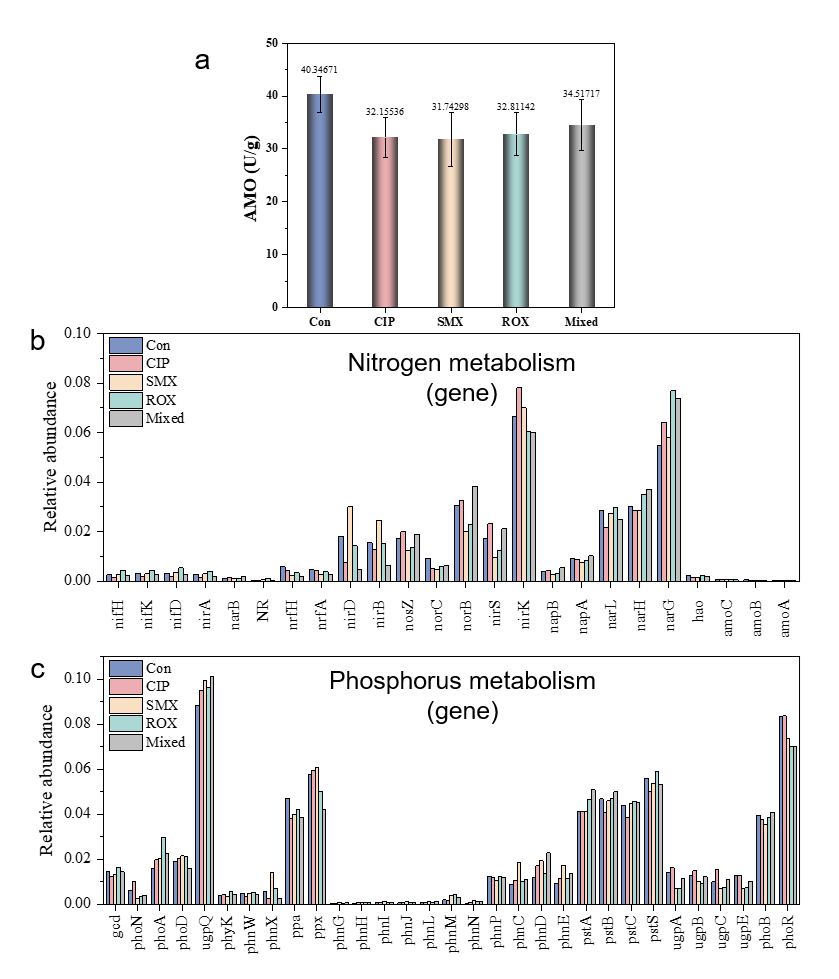


**Fig. S1.** (a) average concentration of ammonia monooxygenase in different groups. Relative abundance patterns of functional genes associated with (b) nitrogen metabolism and (c) phosphorus metabolism under different antibiotic treatments. These results are presented to illustrate overall distribution patterns of functional genes and are not used for quantitative comparison or statistical inference. Relative abundance of functional genes was calculated based on RPKM (reads per kilobase per million mapped reads) normalization of metagenomic data. Gene names are shown according to KEGG Orthology annotations and are presented for descriptive comparison only, without statistical inference.

**Fig. S2.** The average removal efficiency of antibiotics in effluent and the average adsorption amount in sludge under long-term monitoring.


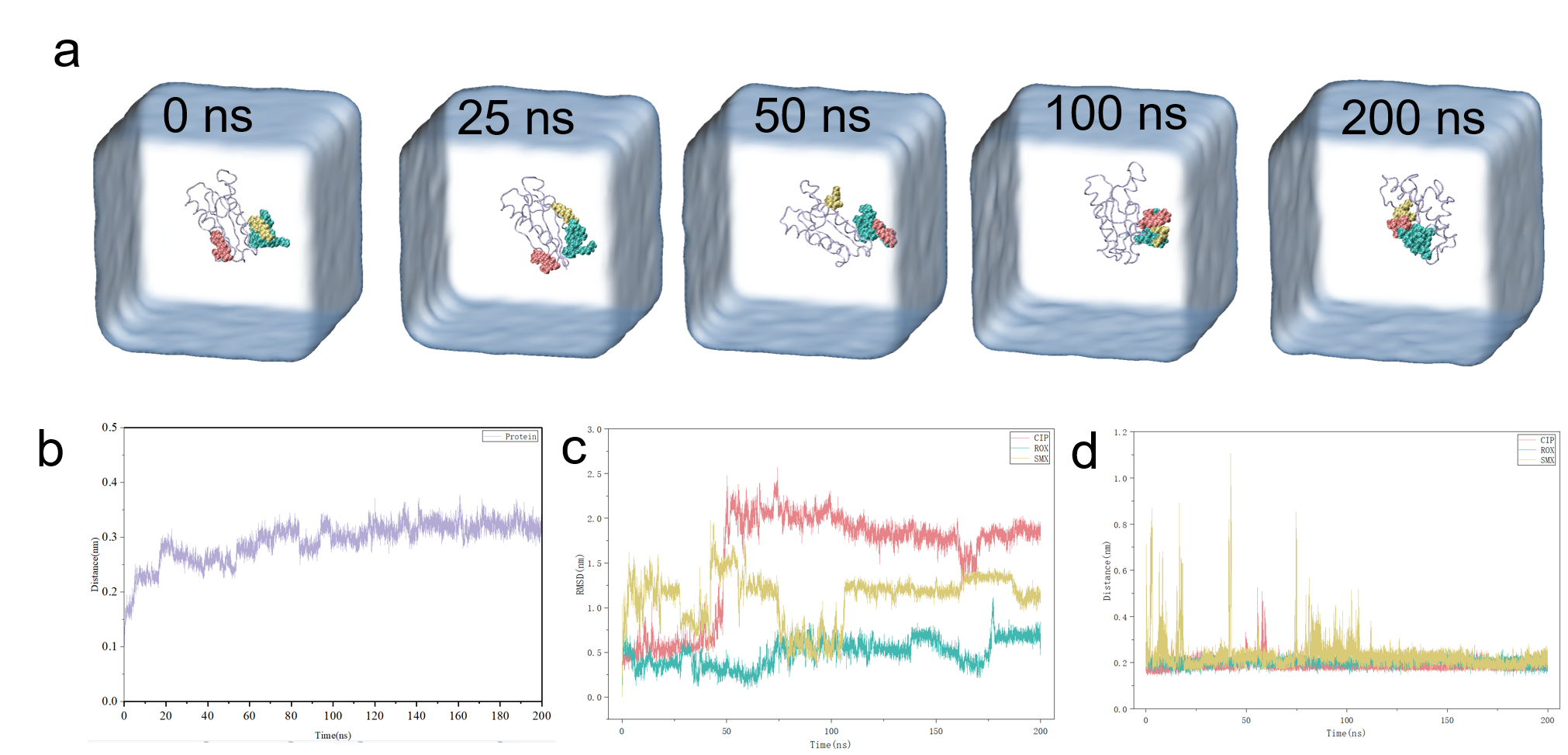


**Fig. S3.** (a) Representative MD snapshots of antibiotic adsorption in the OmpA-antibiotic system; (b) RMSD change of OmpA protein during the complex molecular dynamics simulation process; (c) RMSD change of the antibiotic relative to the OmpA protein during the complex molecular dynamics simulation process; (d) The shortest distance between the antibiotic and OmpA protein during the complex molecular dynamics simulation process.


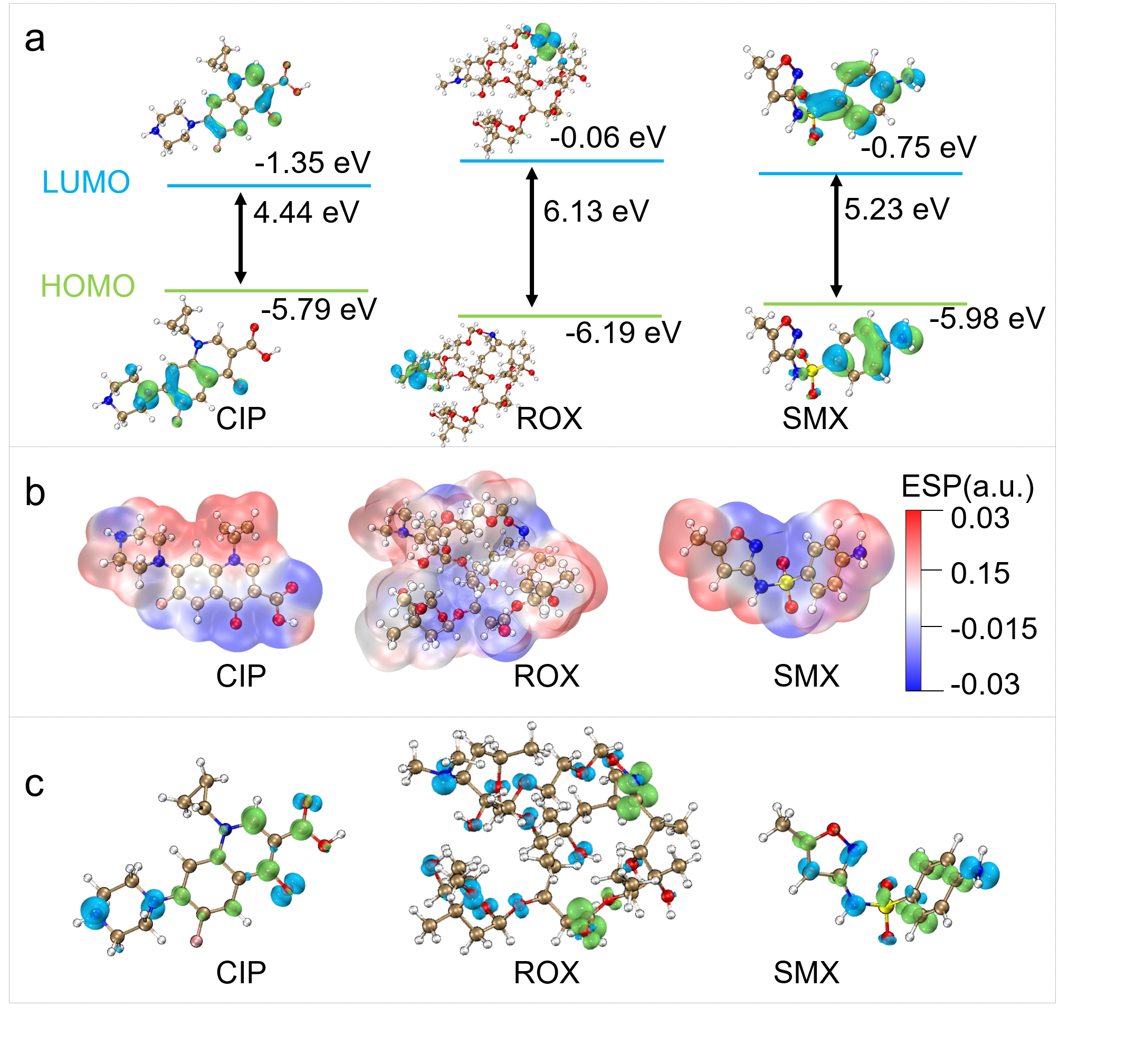


**Fig. S4.** Electronic-structure descriptors for CIP, ROX and SMX: (a) frontier molecular orbitals, (b) electrostatic potential maps, and (c) Fukui functions.


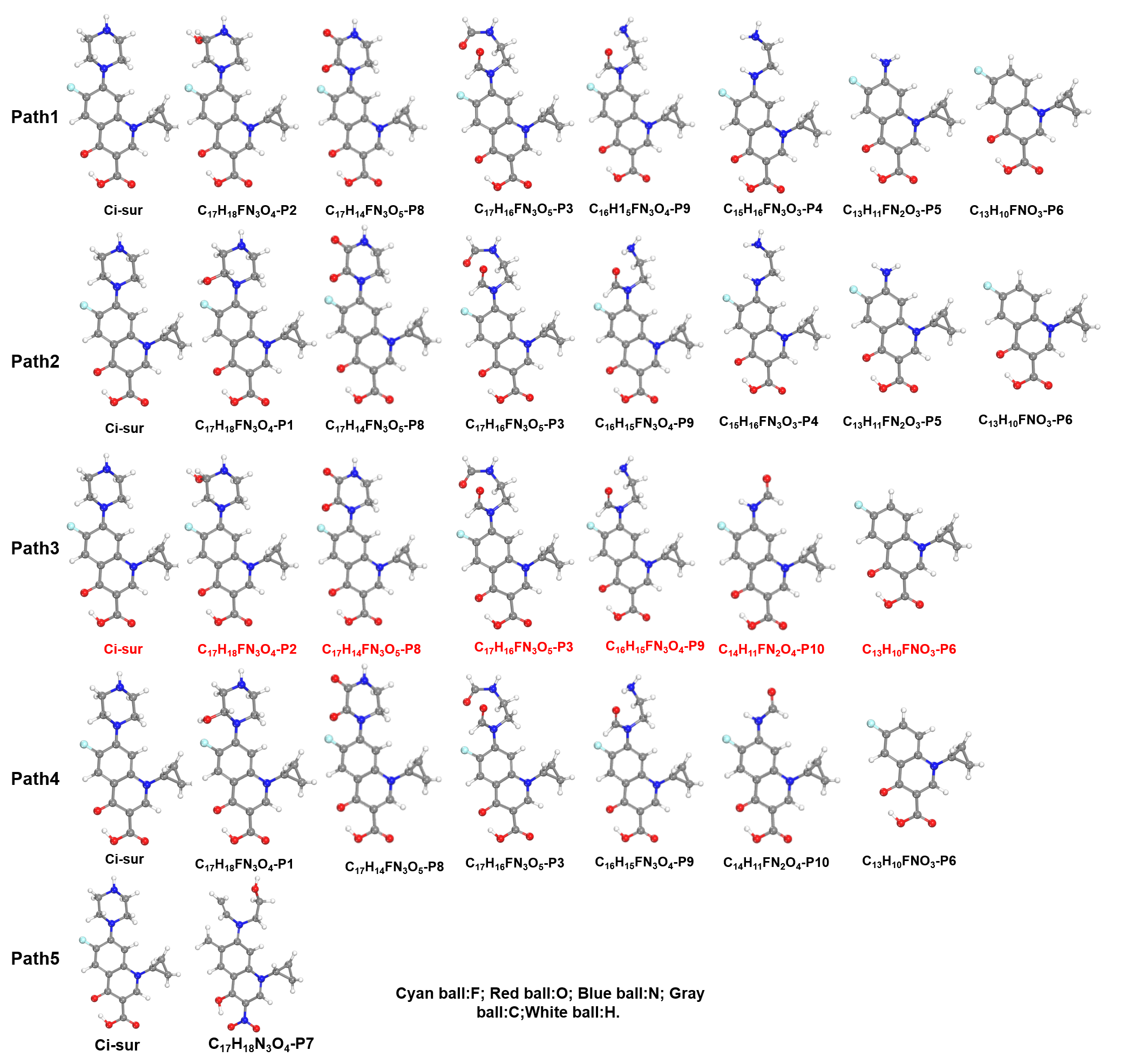


**Fig. S5.** DFT calculation: Proposed biodegradation pathways and product structures for ciprofloxacin.


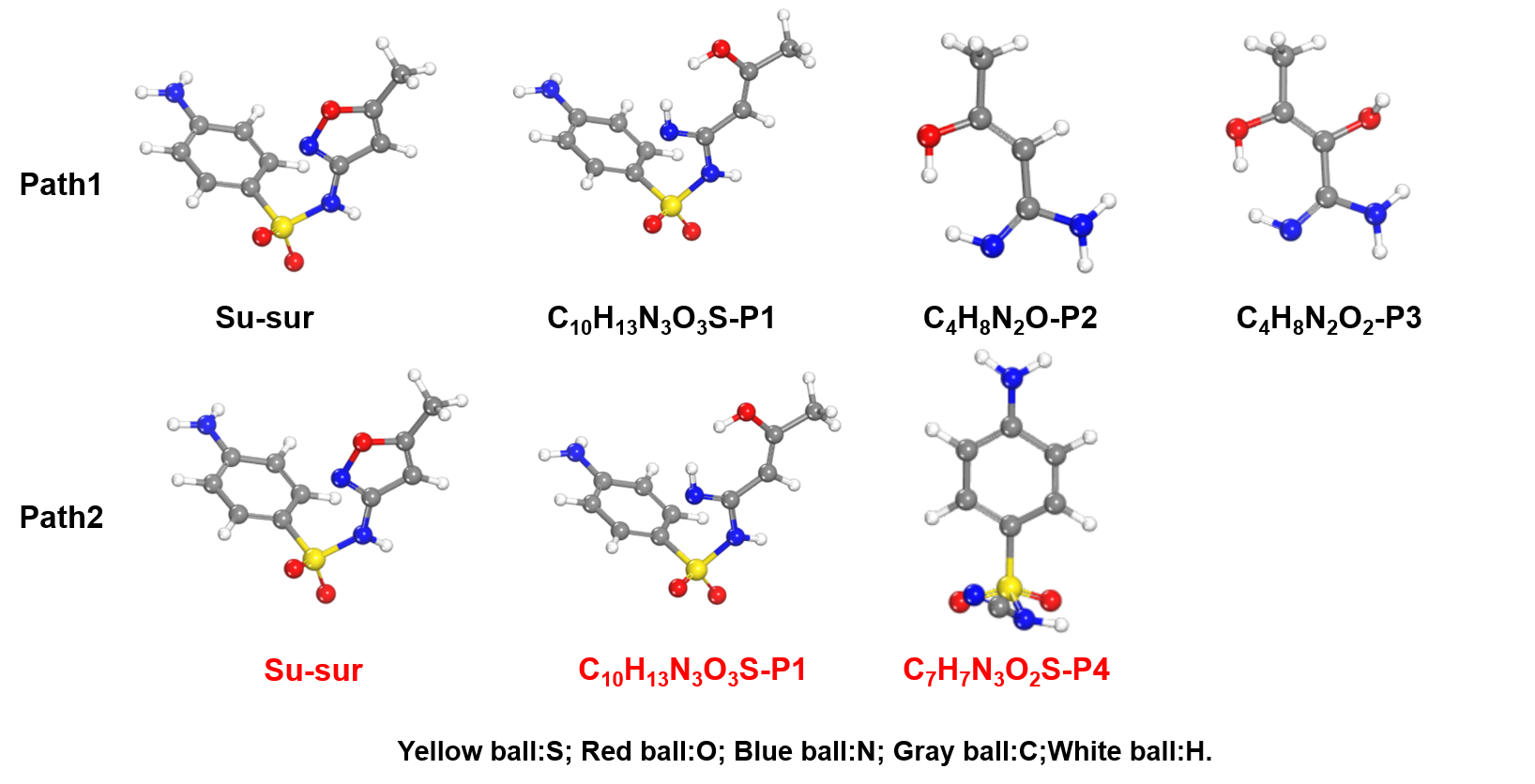


**Fig. S6.** DFT calculations: Proposed biodegradation pathways and product structures for sulfamethoxazole.


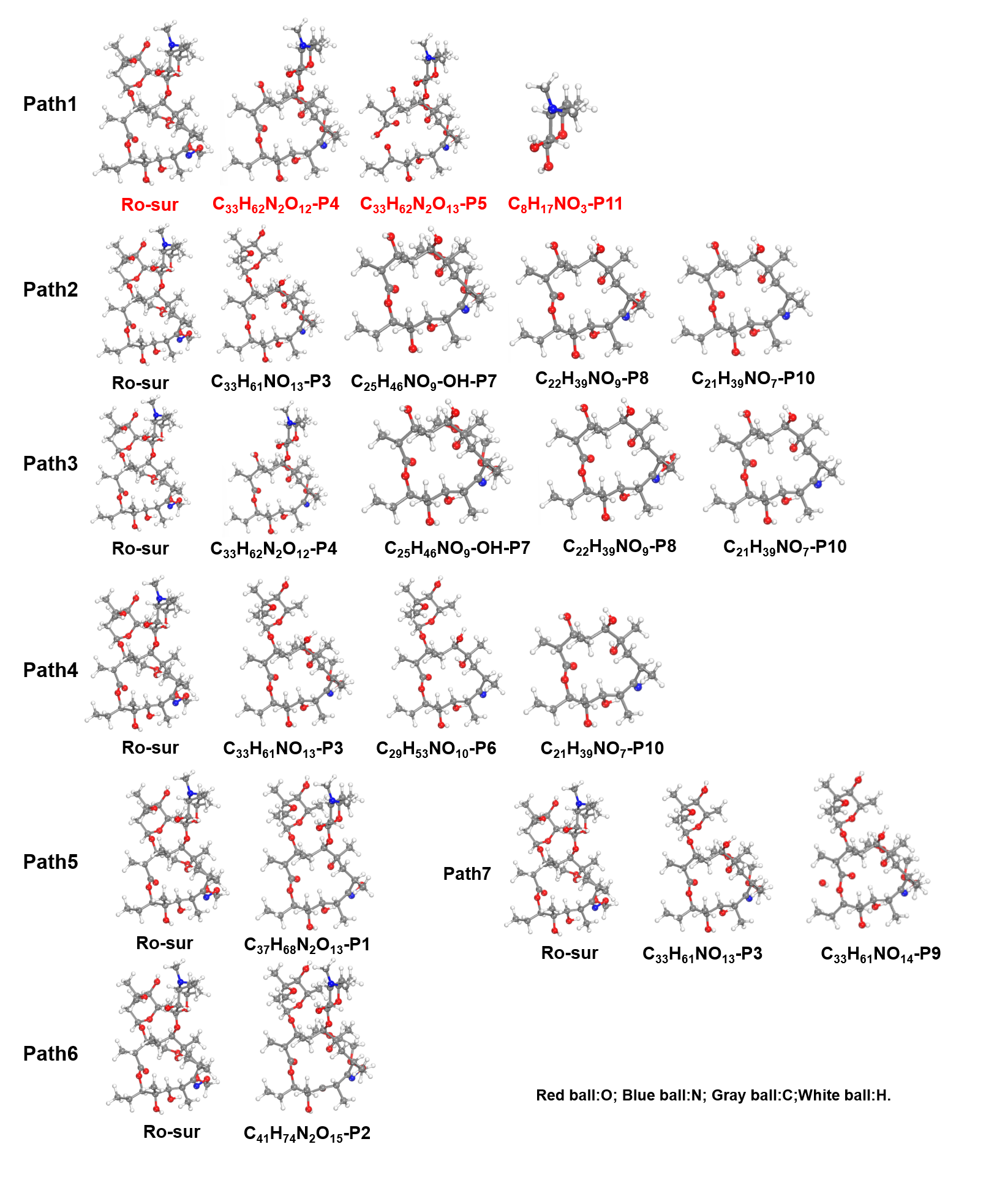


**Fig. S7.** DFT calculations: Proposed biodegradation pathways and product structures for roxithromycin.


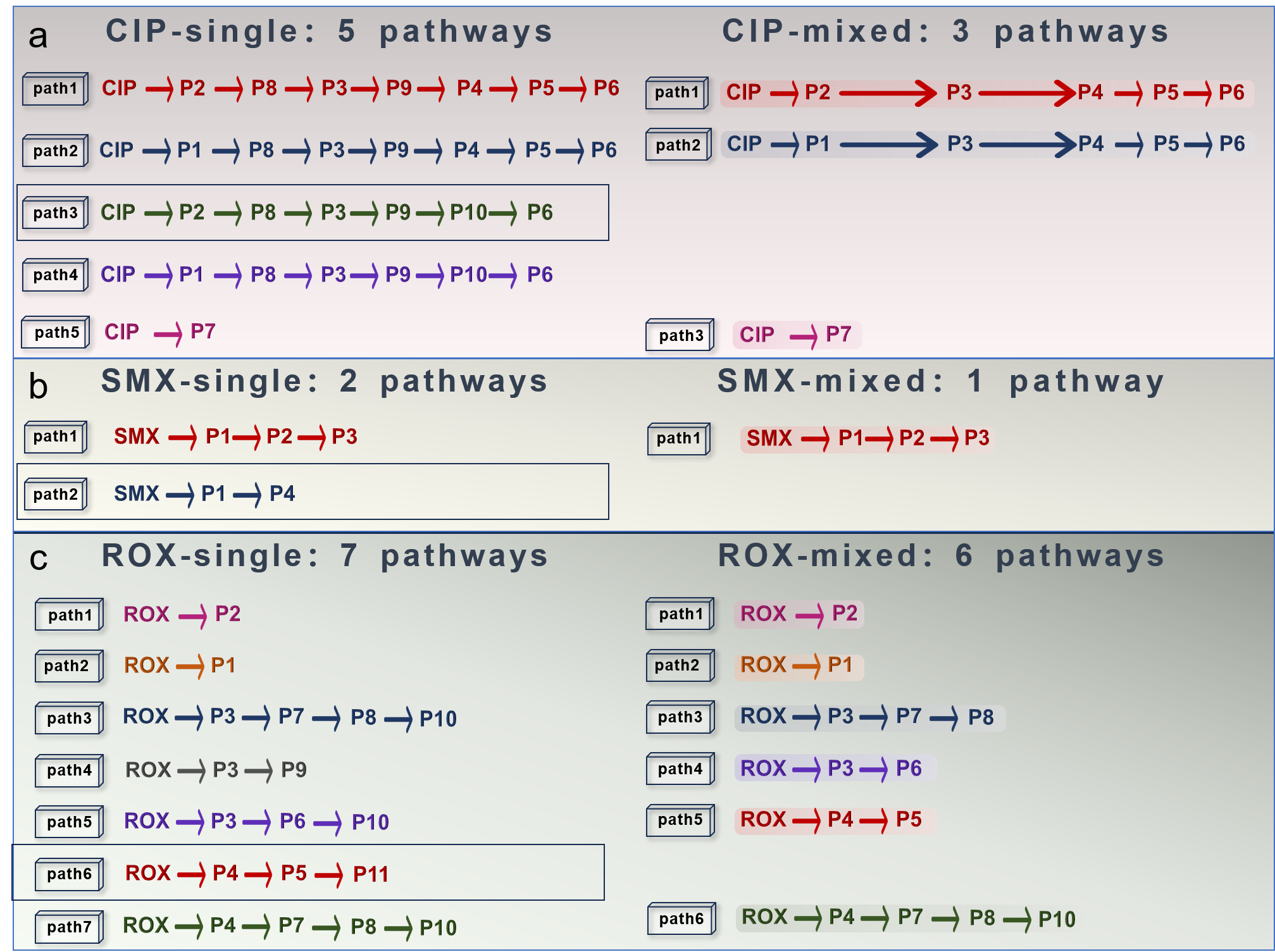


**Fig. S8.** Summary of the degradation pathways for the antibiotics in the single- and mixed-antibiotic groups. The most favorable pathway is highlighted by a box for clarity.


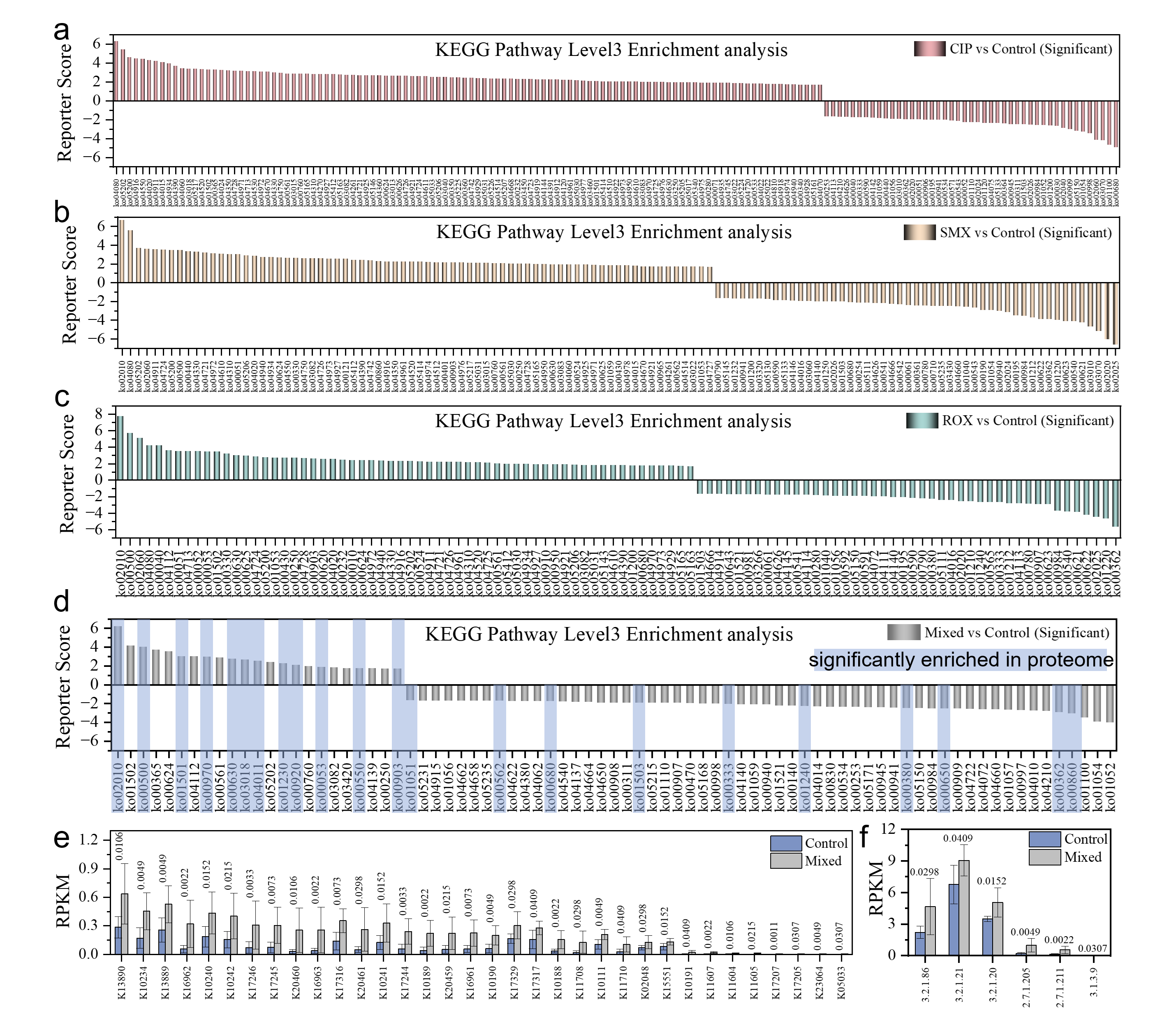


**Fig. S9.** (a-d) Significantly different pathways in the (a) CIP, (b) SMX, (c) ROX, and (d) Mixed groups compared to the control group. (e) Significantly upregulated KOs in the Mixed group compared to the control group within pathway ko02010. (f) Significantly upregulated enzymes in the Mixed group compared to the control group within pathway ko00500.

**
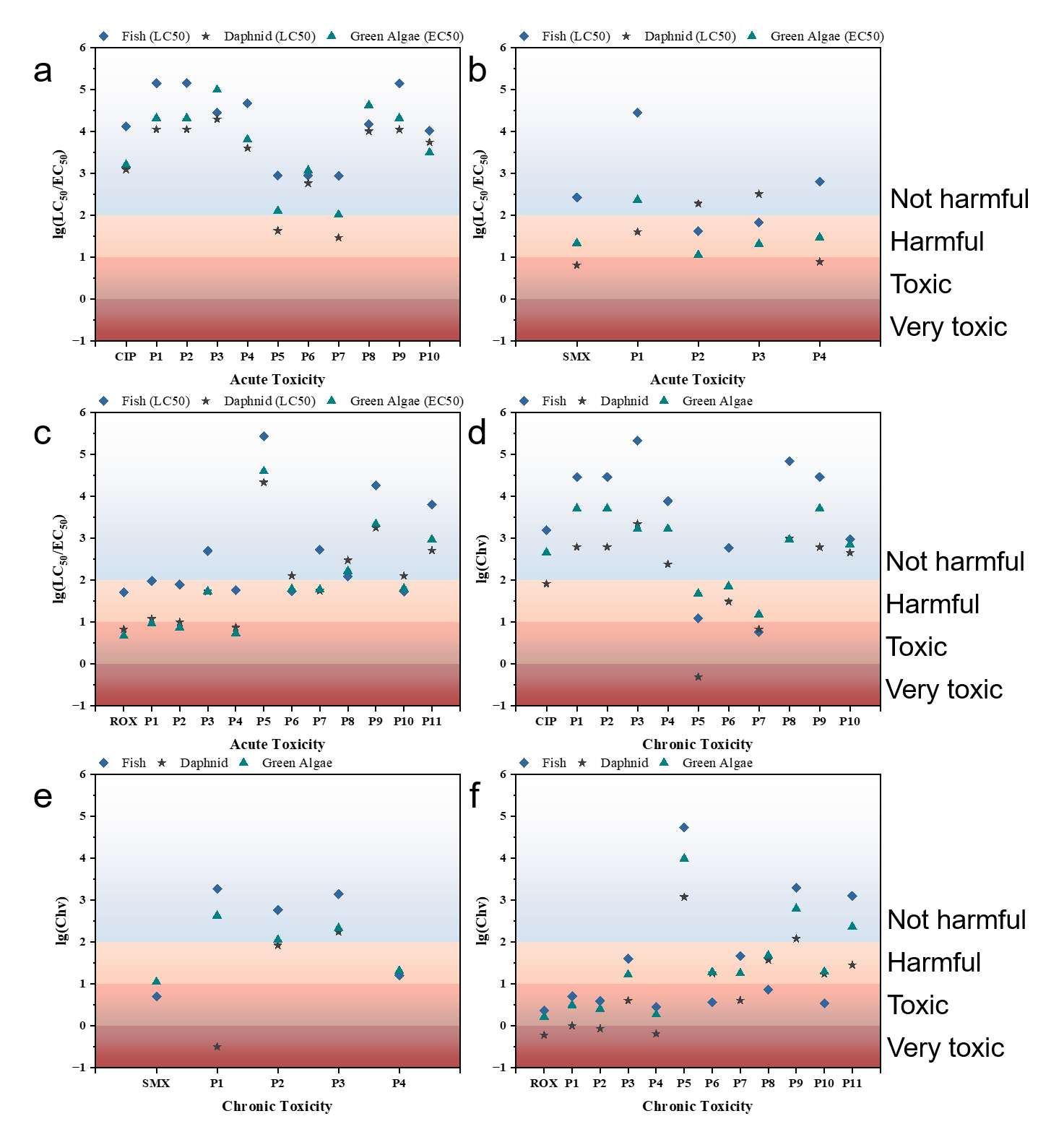
**
**Fig. S10.** Predicted acute toxicity (a–c) and chronic toxicity (d–f) of identified transformation products derived from ciprofloxacin (CIP), sulfamethoxazole (SMX), and roxithromycin (ROX), estimated using the ECOSAR model for representative aquatic organisms.


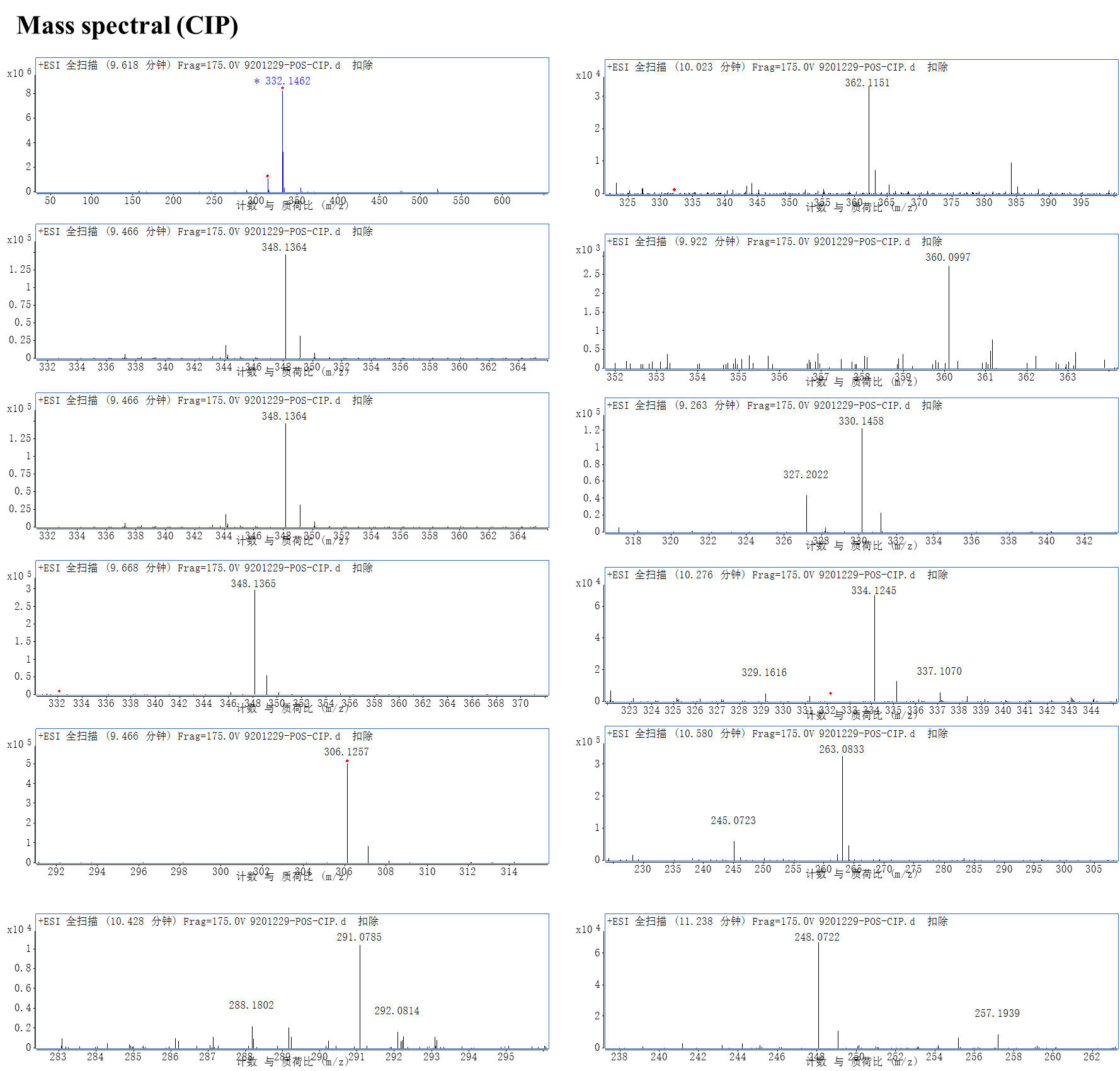


**Fig. S11.** Mass spectral of CIP in single-CIP group.


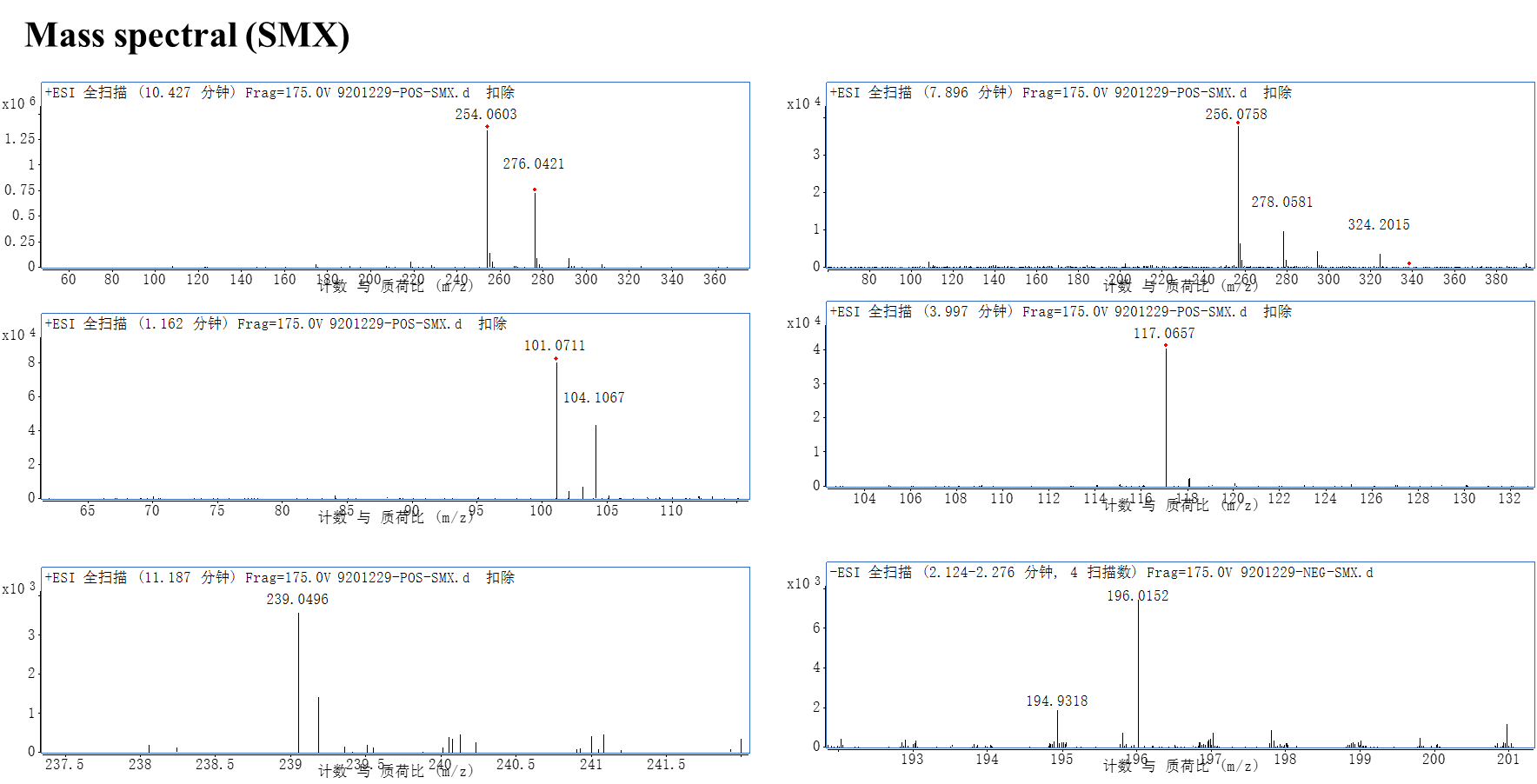


**Fig. S12.** Mass spectral of SMX in single-SMX group.


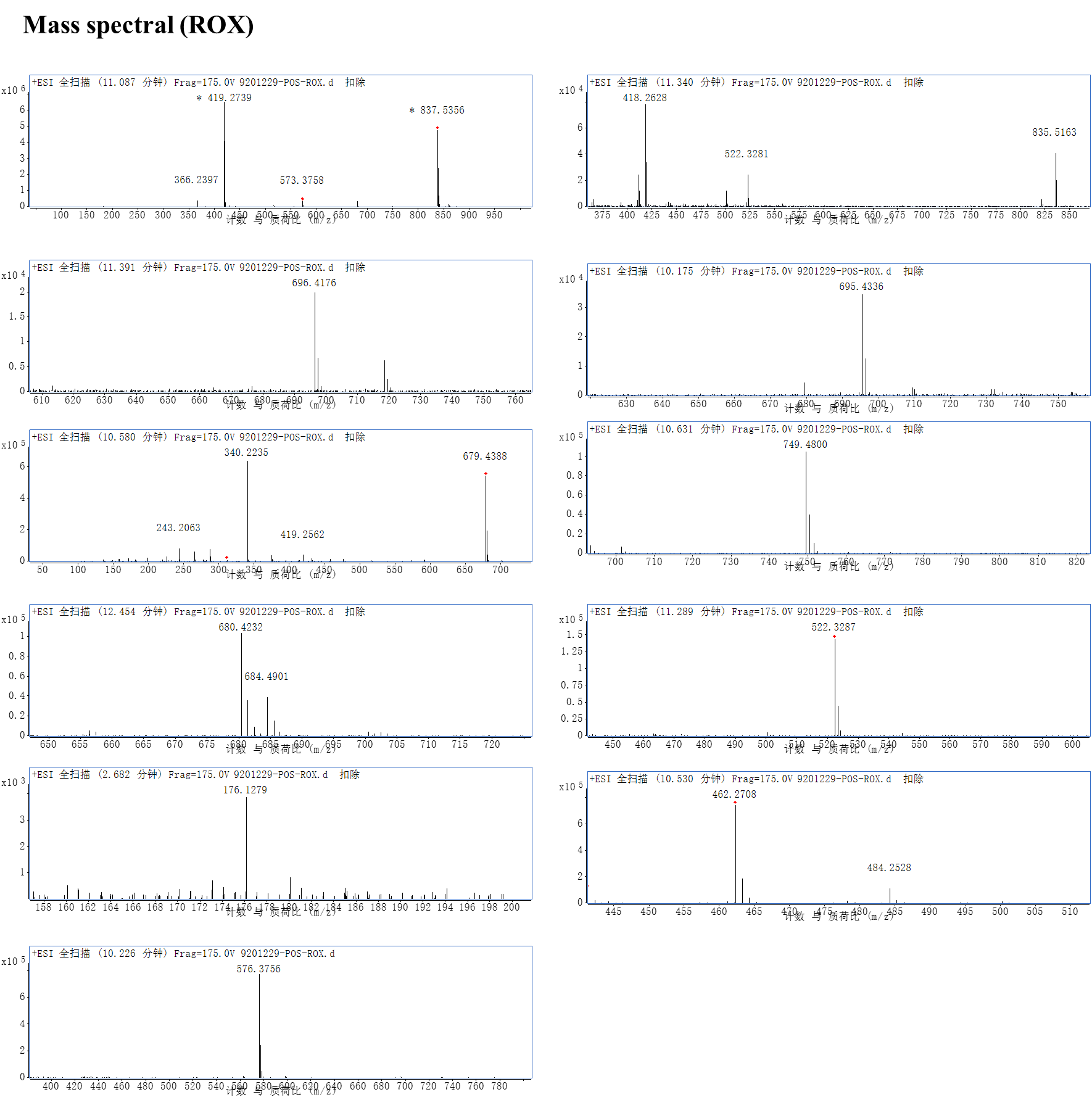


**Fig. S13.** Mass spectral of ROX in single-ROX group.


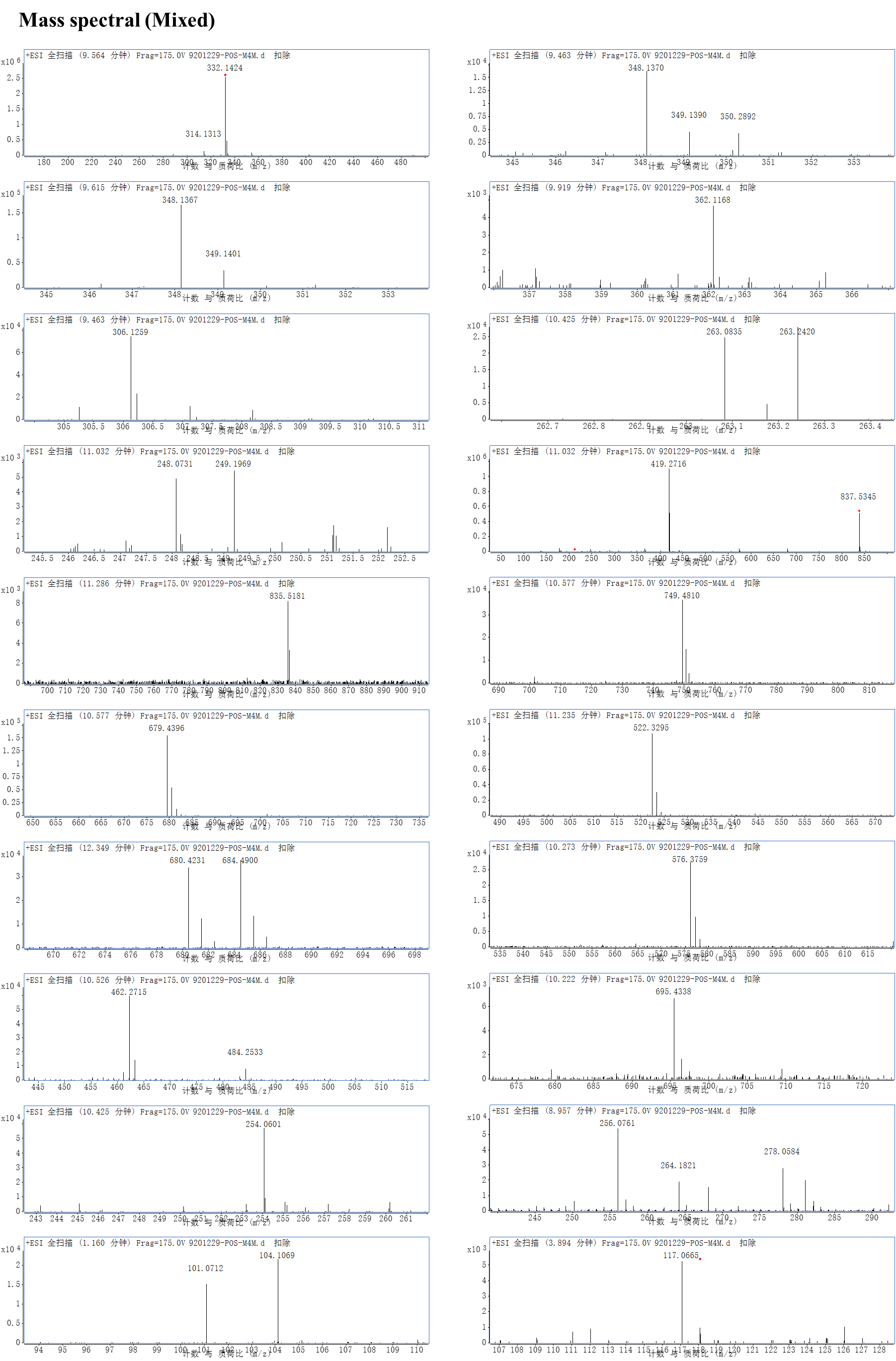


**Fig. S14.** Mass spectral of CIP, SMX and ROX in mixed group.

**Table S1** Reactor Operating Parameters Details

| Time Period | Stage | Duration(min) | Remarks |
| --- | --- | --- | --- |
| 8:30-9:00 | inlet | 30 | 2L |
| 9:00-12:00 | mix | 180 |  |
| 12:00-18:00 | aeration | 360 |  |
| 18:00-20:00 | settle | 120 |  |
| 20:00-20:30 | outlet | 30 | 2L |
| 20:30-21:00 | inlet | 30 | 2L |
| 21:00-0:00 | mix | 180 |  |
| 0:00-6:00 | aeration | 360 |  |
| 6:00-8:00 | settle | 120 |  |
| 8:00-8:30 | outlet | 30 | 2L |

**Table S2** Composition and concentration of synthetic wastewater influent for reactor

| Chemical | Concentration(mg/L) |
| --- | --- |
| Sodium acetate | 100 |
| Glucose | 200 |
| Sucrose | 100 |
| Ammonium chloride | 150 |
| Potassium dihydrogen phosphate | 30 |
| Calcium chloride | 7.4 |
| Magnesium sulfate | 7.5 |
| Iron(III)chloride | 1.45 |
| Iron(II)sulfate | 1.45 |
| Manganese chloride | 0.28 |
| Zinc sulfate | 0.45 |
| Copper sulfate | 0.4 |
| Cobalt chloride | 0.4 |

**References**

[1] G. Kresse, J. Hafner, Ab initio molecular-dynamics simulation of the liquid-metal--amorphous-semiconductor transition in germanium, Physical Review B 49(20) (1994) 14251-14269. <https://doi.org/10.1103/PhysRevB.49.14251>.

[2] K. Hoshino, F. Shimojo, Ab initio molecular dynamics for expanded and compressed liquid alkali metals, Journal of Physics: Condensed Matter 8(47) (1996) 9315. <https://doi.org/10.1088/0953-8984/8/47/022>.

[3] P.E. Blöchl, Projector augmented-wave method, Physical Review B 50(24) (1994) 17953-17979. <https://doi.org/10.1103/PhysRevB.50.17953>.

[4] J.P. Perdew, K. Burke, M. Ernzerhof, Generalized Gradient Approximation Made Simple, Physical Review Letters 77(18) (1996) 3865-3868. <https://doi.org/10.1103/PhysRevLett.77.3865>.

[5] G. Kresse, D. Joubert, From ultrasoft pseudopotentials to the projector augmented-wave method, Physical Review B 59(3) (1999) 1758-1775. <https://doi.org/10.1103/PhysRevB.59.1758>.

[6] H.J. Monkhorst, J.D. Pack, Special points for Brillouin-zone integrations, Physical Review B 13(12) (1976) 5188-5192. <https://doi.org/10.1103/PhysRevB.13.5188>.

[7] M. Orsi, Molecular dynamics simulation of humic substances, Chemical and Biological Technologies in Agriculture 1(1) (2014) 10. <https://doi.org/10.1186/s40538-014-0010-4>.

[8] F. Neese, Software update: the ORCA program system, version 4.0, WIREs Computational Molecular Science 8(1) (2018) e1327. <https://doi.org/https://doi.org/10.1002/wcms.1327>.

[9] T. Lu, F. Chen, Multiwfn: A multifunctional wavefunction analyzer, Journal of Computational Chemistry 33(5) (2012) 580-592. <https://doi.org/https://doi.org/10.1002/jcc.22885>.

[10] <http://sobereva.com/soft/Sobtop/>. <http://sobereva.com/soft/Sobtop/>.

[11] M.J. Abraham, T. Murtola, R. Schulz, S. Páll, J.C. Smith, B. Hess, E. Lindahl, GROMACS: High performance molecular simulations through multi-level parallelism from laptops to supercomputers, SoftwareX 1-2 (2015) 19-25. <https://doi.org/https://doi.org/10.1016/j.softx.2015.06.001>.
